# Splicing-coupled 3′ end formation requires a terminal splice acceptor site, but not intron excision

**DOI:** 10.1093/nar/gkt446

**Published:** 2013-05-28

**Authors:** Lee Davidson, Steven West

**Affiliations:** Wellcome Trust Centre for Cell Biology, Institute for Cell Biology, University of Edinburgh Michael Swann Building, King’s Buildings, Mayfield Road, Edinburgh EH9 3JR, United Kingdom

## Abstract

Splicing of human pre-mRNA is reciprocally coupled to 3′ end formation by terminal exon definition, which occurs co-transcriptionally. It is required for the final maturation of most human pre-mRNAs and is therefore important to understand. We have used several strategies to block splicing at specific stages *in vivo* and studied their effect on 3′ end formation. We demonstrate that a terminal splice acceptor site is essential to establish coupling with the poly(A) signal in a chromosomally integrated β-globin gene. This is in part to alleviate the suppression of 3′ end formation by U1 small nuclear RNA, which is known to bind pre-mRNA at the earliest stage of spliceosome assembly. Interestingly, blocks to splicing that are subsequent to terminal splice acceptor site function, but before catalysis, have little observable effect on 3′ end formation. These data suggest that early stages of spliceosome assembly are sufficient to functionally couple splicing and 3′ end formation, but that on-going intron removal is less critical.

## INTRODUCTION

Most human pre-mRNAs contain multiple introns that are removed by splicing. The splicing process has two catalytic steps with the first releasing the 5′ exon and the second joining the two exons together with consequent release of an intron lariat (1). It is performed by a set of five small nuclear RNAs (snRNAs) in concert with 100 s of protein factors. Intronic sequences, such as the 5′ and 3′ splice site, intervening branch-point and polypyrimidine tract, play a vital role (2). The function of snRNAs in splicing has been well characterized through decades of study using both *in vitro* and *in vivo* systems. First, U1 snRNA (U1) contacts the 5′ splice site after which U2 snRNA (U2) is recruited to the branch-point following recognition of the 3′ splice site and polypyrimidine tract by U2AF35 and 65, respectively. U4, U5 and U6 snRNAs then join, and two-step catalysis occurs after U1 and U4 are ejected from the spliceosome (2). Splicing of most introns in higher eukaryotes is thought to occur by definition of the comparatively shorter exons, across which 3′ and 5′ splice sites are recognized by U2 and U1, respectively (3).

The majority of pre-mRNAs undergo cleavage and polyadenylation at their 3′ ends. This process requires a poly(A) (pA) signal within the RNA, which is composed of a hexameric sequence, usually AAUAAA, followed by a U or G/U rich sequence (4,5). Cleavage of the transcript occurs between these two elements and is carried out by the CPSF73 endonuclease, which is part of the Cleavage and Polyadenylation Specificity Factor (CPSF) complex (6). Other protein complexes including Cleavage Stimulation Factor (CstF), Cleavage Factors I and II (CFIm and CFIIm) are also required (7). The upstream cleavage product is polyadenylated to form the mRNA, whereas the downstream product is rapidly degraded in a process linked to termination of RNA polymerase II (Pol II) (8,9).

First observed 30 years ago (10), it is now established that there is reciprocal functional coupling between splicing and 3′ end processing of RNA (11,12). This is through definition of the last exon, which requires a terminal splice acceptor (tSA) site and a pA signal. Mutation of either sequence disrupts both splicing and 3′ end formation as well as the linked process of transcriptional termination (12). In addition, trans-acting factors play a role by forming bridging contacts across the terminal exon. Prominent among these are U2AF65, which binds cleavage factor Im and poly(A) polymerase (13,14), and SF3b components, which interact with the CPSF complex (15). A large-scale purification of the 3′ end processing complex identified other splicing factors that may function analogously (16).

3′ end formation is also negatively regulated by certain splicing factors. Early experiments showed that 5′ splice site sequences are involved in silencing some viral pA signals through the recruitment of U1 snRNA (17–20). More recently, U1 was found to suppress the processing of a huge number of pA signals embedded within human pre-mRNAs (21,22). There must therefore be a mechanism of co-ordinating this suppression with activation by later splicing factors to establish coupling with 3′ end formation.

The complex regulation of 3′ end formation by splicing should be considered in the context of transcription, to which both reactions are coupled *in vivo* (23). This is achieved by ternary interactions between processing factors, the pre-mRNA transcript and the C-terminal domain of the large subunit of Pol II. Consequently, co-transcriptional splicing of introns is common (24–27). However, introns near to the 3′ end of a transcript frequently display a lower incidence of co-transcriptional removal (25,28,29).

We have used several different approaches to determine how and when splicing is coupled to 3′ end formation. We mutated the tSA site in a chromosomally integrated β-globin gene. This inhibited splicing and 3′ end formation in a manner involving U1 snRNA and was associated with the loss of U2AF recruitment to the tSA site. To further analyse the requirements for tSA site function, we blocked splicing at subsequent stages using the small molecule Spliceostatin A (SSA) and by inhibiting U4 snRNA. These approaches reduced splicing but surprisingly had little effect on 3′ end formation. Thus, a tSA site establishes coupling between splicing and 3′ end formation, which is sufficient for cleavage and polyadenylation even when intron removal is inefficient.

## MATERIALS AND METHODS

### Plasmids

The β-globin plasmid for Figure 5 (βΔ5-7) has been described previously (30) as has the Tat plasmid (31). The VA plasmid contains the adenovirus VAI gene and was a gift from Nick Proudfoot. For the βWT cell line, β-globin and a portion of 3′ flank was amplified from βΔ5-10 (30) using primers βE1F and βFR. This was inserted into the pcDNA5/FRT/TO vector that was initially linearized by PCR with primers pcF and pcR. This new vector (βWT) was used as a template for site-directed mutagenesis with primers tSAmF and tSAmR for the βtSA cell line and with βpAmF and pAmR for the pAmF line. βWTCoTC was made in the same way as βWT, but the insert was generated by PCR amplification of the previously described βΔ5-7 (30) plasmid with βE1F and βFR.

### Cell culture

Cells were grown in Dulbecco’s modified Eagle’s medium supplemented with 10% foetal calf serum. SSA was used at 50 ng/ml unless otherwise stated. Actinomycin D (ActD) was used at 10 μg/ml. Electroporation was performed on a confluent 10-cm diameter dish of cells using 10–15 μM anti-sense morpholino (AMO) in 400 μl of Dulbecco’s modified Eagle’s medium using a 4-mm gap cuvette (960 μF, 280 v in a Biorad gene pulser). RNA was isolated 3 h after electroporation. For transient transfection, 2.5 μg of reporter and 0.5 μg VA were introduced using Xtreme gene (Roche). For anti-sense oligonucleotide (ASO) experiments, 500 pmol of oligo was introduced into a 24-well dish of cells using Lipofectamine RNAiMax (Life Technologies). For all plasmid and ASO transfections, RNA was isolated after 24 h. For stable integration, a 6-cm dish of Flp-IN HEK cells was transfected with 1 μg of the appropriate β-globin plasmid in addition to 3 μg of pOG44 using JetPrime (Polyplus) transfection reagent. Forty-eight h post-transfection, cells were transferred to a tissue culture flask and grown in the presence of Hygromycin (100 μg/ml) and Blasticidin (10 μg/ml) until resistant cells emerged. Transcription was induced by overnight growth in media supplemented with 1 μg/ml tetracycline (tet).

### Antibodies

Pol II (N20, Santa Cruz); anti-CstF77 (Sigma, C0249); anti-U2AF65 (Sigma, U4758).

### RNA isolation

Chromatin-associated and nucleoplasmic RNA was isolated as previously described (32,33). RNA was treated twice with Turbo DNase (Ambion). Fractionation efficiency was determined by detection of endogenous U1. Total RNA was isolated using Trizol (Life Technologies). For real-time PCR analysis, 1 μg of RNA was reverse transcribed using Inprom II (Promega). Parallel reactions were performed in the absence of reverse transcriptase. In all, 1/20th of the cDNA mix was used for real-time PCR using 5–10 pmol of forward and reverse primer and Brilliant III SYBR mix (Agilent Technologies) in a Qiagen Rotorgene machine. Differences were calculated using comparative quantitation.

### Chromatin and RNA Immunoprecipitation and S1 nuclease analysis

Detailed protocols are provided in the supplementary material.

### Primer sequences

Primer, AMO and ASO sequences are provided in the supplementary material.

## RESULTS

### A tSA site is required for maximal 3′ end formation in a chromosomal context

**Figure 1.**
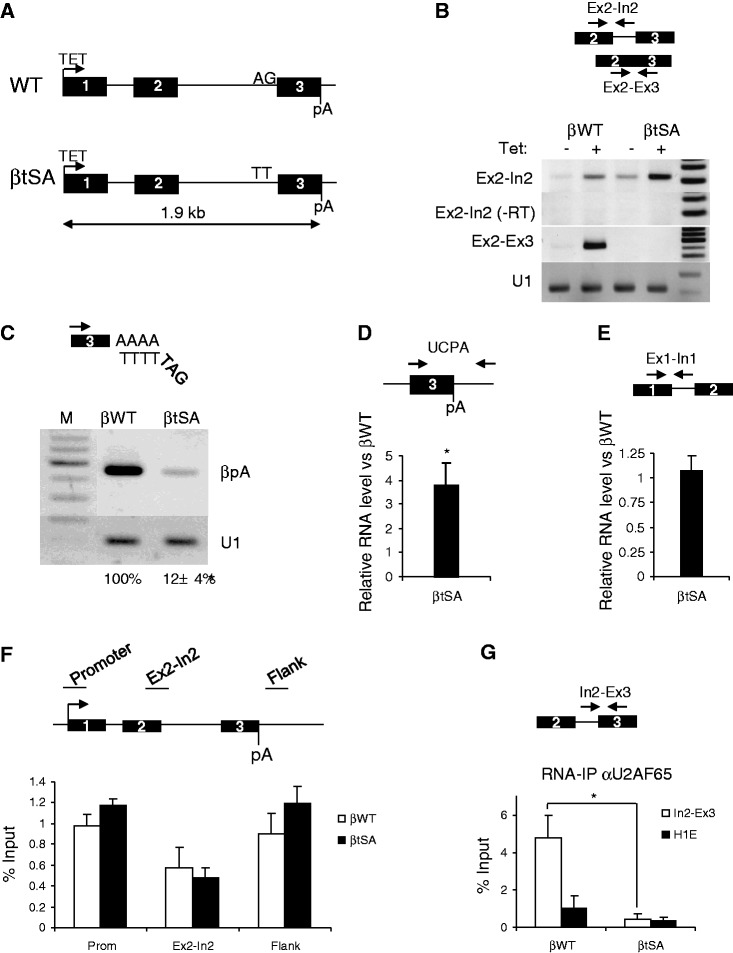
(**A**) Diagram of WT and tSA mutated β-globin genes integrated into the HEK cell genome. The tet-inducible CMV promoter (arrow), exons (numbered boxes) and poly(A) site (pA) are indicated. WT (AG) and mutant (TT) tSA sites are shown. (**B**) Confirmation of tet-inducible transcription of β-globin in βWT and βtSA HEK cells. Agarose gels show, from the top, unspliced intron 2 (Ex2-In2) RNA, a control where reverse transcriptase was excluded, spliced (Ex2-Ex3) and a U1 snRNA loading control. (**C**) 3′ RACE analysis of cleaved and polyadenylated βWT and βtSA RNA. Diagram shows tag primer used for cDNA synthesis and partner primer in exon 3 (black arrow). Agarose gel shows 3′ end-processed β-globin RNA (βpA) and the U1 loading control. Real-time PCR quantitation is shown underneath where βtSA values are given as a percentage of βWT. (**D**) Real-time PCR of non-pA cleaved βWT and βtSA transcripts. Diagram shows PCR primers that span the pA site. Graph shows quantitation where the levels in βtSA cells are plotted as a fold change relative to those in βWT cells (set to 1 following normalisation to U1 levels). (**E**) Real-time PCR of exon1–intron 1 RNA in βWT and βtSA samples. Diagram shows PCR primers that span the 5′ splice site of intron 1. Graph shows quantitation where the levels in βtSA cells are plotted as a fold change relative to those in βWT cells (set to 1 following normalization to U1 levels). (**F**) ChIP analysis of Pol II loading on βWT and βtSA genes. Diagram shows β-globin gene and positions of PCR amplicons. Graph shows the percentage of DNA immunoprecipitated. (**G**) RIP analysis of U2AF65 binding to the tSA site of β-globin pre-mRNA in βWT or βtSA lines. Quantitation is expressed as percentage input. Values obtained from Histone H1E (used as a negative control) are also shown. Error bars show standard deviation (SD) from at least three biological repeats. Astrisk denotes *P* < 0.05 for βtSA versus βWT.

To analyse coupling between splicing and 3′ end formation in a chromosomal context, we constructed two HEK cell lines containing either a single stably integrated wild-type (βWT) β-globin gene or one with a mutated tSA site (βtSA) (Figure 1A). Transcription was under the control of a tet-inducible CMV promoter. To verify the two cell lines, total RNA was isolated from βWT or βtSA cells after overnight growth in media supplemented or not with tet. Random hexamer generated cDNA was analysed by PCR to detect unspliced (Ex2-In2) and spliced (Ex2-Ex3) β-globin transcripts (Figure 1B). Unspliced RNA was detected in both the βWT and βtSA lines, and levels were increased following tet induction. There was a higher level of unspliced intron 2 in βtSA samples in line with the expected splice inhibitory effect of the mutation. Correctly spliced β-globin was detected only in samples from βWT cells, again displaying a tet-dependent increase. Endogenous β-globin is not expressed in HEK cells (34).

To test whether the βtSA mutation affected 3′ end formation in the chromosomal context as it does in plasmids (12), we performed 3′ RACE analysis on total RNA from tet-induced βWT and βtSA cells (Figure 1C). A tagged oligo-dT was used for reverse transcription, and subsequent PCR was done with primers directed to the tag and to exon 3 to detect all polyadenylated species. A strong band was detected in the βWT sample, but a much weaker band was detected in βtSA cells indicating little processing in that case (∼8-fold less than βWT). We also analysed random hexamer generated cDNA using primers spanning the pA site to detect β-globin transcripts that were not 3′ end processed (Figure 1D). The tSA site mutation resulted in a 3–4-fold accumulation of this species compared with βWT samples, which is again consistent with reduced 3′ end formation. This was also associated with a mild reduction in the recruitment of CstF77 pA-processing factor to the βtSA gene (Supplementary Figure S1).

As pre-mRNA processing defects often reduce transcription (35–37), we tested whether this was the case on βtSA. There was little difference in the abundance of unspliced intron 1 in βWT and βtSA cell lines (Figure 1E), which we used as a proxy for transcriptional activity. We also analysed Pol II loading on the βWT- and βtSA-mutated β-globin genes using chromatin immunoprecipitation (ChIP) (Figure 1F). This was assessed at three positions across the gene using an antibody that detects all forms of Pol II (N20). No significant differences were observed over any of the regions between the two cell lines, indicating that similar levels of Pol II were present. Collectively, these data demonstrate that the tSA site mutation inhibits splicing and 3′ end processing, which is not mediated through obvious reductions in transcription in this context.

Mutation of a tSA site is also predicted to prevent the recruitment of relevant 3′ splice site-binding factors that participate in terminal exon definition. We tested this by performing RNA immunoprecipitation (RIP) to analyse binding of the 65 kDa component of U2AF, U2AF65, to the tSA site of βWT or βtSA transcripts. Immunoprecipitated RNA was reverse transcribed with random hexamers and then PCR amplified with primers across the tSA site. Histone H1E mRNA was used as a negative control, as U2AF65 is not expected to bind this transcript. A 4–5-fold greater proportion of βWT tSA site-containing transcripts were recovered in comparison (Figure 1G). In contrast, equivalent βtSA transcripts were close to this background level, suggesting that U2AF is poorly recruited in the absence of a tSA site. This is in line with previous observations that the splice site AG dinucleotide is important for its efficient association with RNA (38,39). Its absence from βtSA transcripts correlates with their poor processing and with data from several laboratories showing U2AF65 to promote cleavage and polyadenylation (13,40,41).

### 3′ end processing of βtSA pre-mRNA is activated by inhibition of U1 snRNA

**Figure 2.**
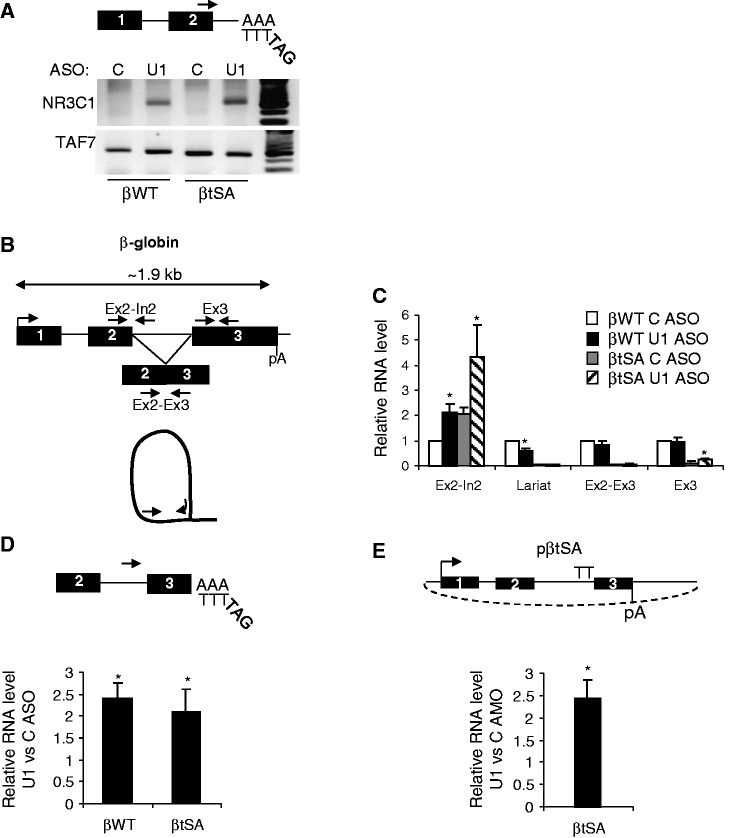
(**A**) Diagram shows 3′ RACE detection of premature 3′ end formation within intron 2 of the NR3C1 transcript. Top panel shows this processed product in βWT or βtSA cells transfected with control or U1 ASO. Lower panel shows intronless TAF7 loading control. (**B**) Diagram depicting primers used for quantitative RT-PCR analysis of β-globin transcripts. The β-globin gene is shown along with primers (black arrows) to detect unspliced intron 2 (Ex2-In2), spliced (Ex2-Ex3), total (Ex3) as well as the excised lariat intron 2. (**C**) Real-time PCR quantitation of Ex2-In2, Lariat, Ex2-Ex3 and Ex3 RNA in total RNA from βWT and βtSA HEK cells transfected with control or U1 ASO. Values are expressed as a fold-change relative to control ASO-treated βWT (given a value of 1 after normalizing to HistH1E). (**D**) Quantitative 3′ RACE analysis of β-globin RNA from samples isolated from control or U1 ASO-treated βWT and βtSA HEK cells. Diagram shows primers used to detect the unspliced 3′ end processed product. For both the βWT and βtSA experiments, the value obtained in U1 ASO samples is expressed as a fold increase over that obtained in control samples (given a value of 1 in each case after normalizing to HistH1E). (**E**) Quantitative 3′ RACE analysis of unspliced 3′ end processed RNA from HeLa cells transiently transfected with a βtSA plasmid and electroporated with control or U1 AMO. Values are expressed as a fold increase as compared control AMO-treated samples (given a value of 1 after normalizing to HistH1E). Error bars show SD from at least three biological repeats. Asterisk denotes where *P* < 0.05 for U1 AMO/ASO-dependent changes.

In addition to blocking the recruitment of factors that may act positively in 3′ end formation, a tSA site mutation may also leave the pA signal vulnerable to negative effects. In particular, U1 inhibits cleavage and polyadenylation at pA sites when bound to nearby 5′ splice sites (17,21,22,42), which are all intact in βtSA transcripts. To test whether this was the case, we inhibited the ability of U1 to interact with pre-mRNA using an ASO that binds to its 5′ end (43,44). Treatment with the ASO is therefore predicted to block the earliest steps in spliceosome assembly. Transfection of the U1 ASO into βWT and βtSA HEK cells induced premature 3′ end formation within the NR3C1 pre-mRNA, which is a known consequence of U1 inhibition and confirms the effectiveness of the approach (22) (Figure 2A).

To determine the effect of U1 inhibition on β-globin splicing, reverse transcription and real-time PCR were used to quantitate the level of unspliced intron 2 (Ex2-In2), intron 2 lariat, spliced (Ex2-Ex3) and total (Ex3) β-globin transcripts (Figure 2B and C). For βWT cells, U1 ASO caused a significant increase in unspliced intron 2 and a decrease of lariat intron 2 consistent with splicing inhibition. Little change in total or spliced βWT RNA was seen following U1 ASO treatment potentially because these species are stable or because some splicing still occurs. For βtSA cells, U1 ASO treatment caused an increase in amounts of unspliced intron 2 and exon 3, but spliced transcripts remained at low levels as expected.

To test for the effect of depleting U1 binding on 3′ end formation, we quantitatively analysed unspliced pre-mRNA that was 3′ end processed (Figure 2D). To assay RNAs for which splicing was inhibited, RNA from control or U1 ASO-treated cells was reverse transcribed with a tagged oligo-dT probe and then amplified using the tag primer and a primer within intron 2. Only authentic 3′ end-processed RNA is detected by this approach, as it is absent when the pA signal is mutated (Supplementary Figure S2). U1 inhibition caused a 2-fold increase of this species in βWT cells, suggesting that 3′ end formation occurs when U1 is not recruited to the natural intron. Interestingly, a similar effect on 3′ end processing was observed in βtSA cells. Furthermore, an AMO targeting U1 gave the same result in βtSA plasmid transfected HeLa cells treated for only 3 hours (Figure 2E). This enhancement of 3′ end formation of βtSA transcripts strongly suggests that bound U1 normally plays a role in suppressing cleavage and polyadenylation in this situation. This is consistent with the confirmed function of U1 bound to 5′ splice site sequences, which are all intact in βtSA, in inhibiting cleavage and polyadenylation (21,22,42).

### 3′ end formation occurs in the presence of SSA in a tSA site-dependent manner

The data so far show that 3′ end formation requires a tSA site and that, in part, this is to alleviate the negative effects of U1. We were next interested in testing whether a block to splicing that occurs after tSA site recognition would impair 3′ end formation. SSA was used, as it blocks splicing after U1 and U2 have bound the RNA but before catalysis (45–47). Unlike the tSA site mutation, SSA does not affect the recruitment of U2AF65 (47,48), suggesting that recognition of the 3′ splice site still occurs. As SSA blocks splicing before catalysis, this experiment also tests whether intron excision plays a role in coupling splicing and 3′ end formation.

**Figure 3.**
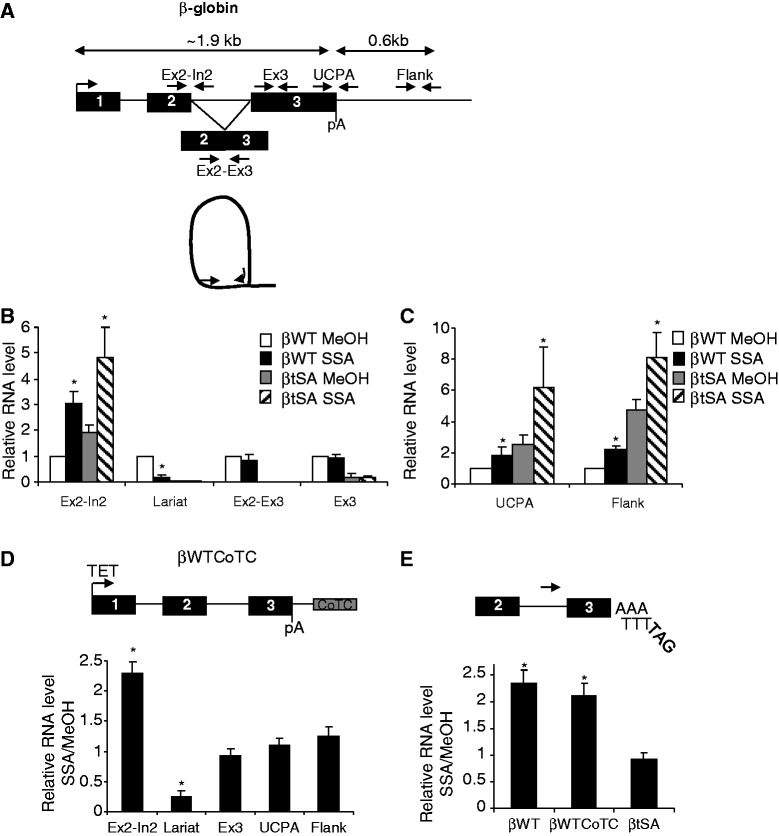
(**A**) Diagram depicting primers used for quantitative RT-PCR analysis of β-globin transcripts. Labels are as with Figure 2B with the addition of, non-pA cleaved (UCPA) and flank primers. (**B**) Real-time PCR quantitation of Ex2-In2, Lariat, Ex2-Ex3 and Ex3 RNA in total RNA from βWT and βtSA HEK cells treated with MeOH or SSA. Following normalization to U1 levels, values are expressed as a fold change relative to those obtained in WT MeOH treated samples (given a value of 1). (**C**) Real-time PCR quantitation of non-pA-cleaved and flank RNA in total RNA from βWT and βtSA HEK cells treated with MeOH or SSA. Following normalization to U1 levels, values are expressed as a fold change relative to those obtained in βWT MeOH-treated samples (given a value of 1). (**D**) Real-time PCR quantitation of Ex2-In2, lariat, Ex3, UCPA and flank RNA in βWTCoTC HEK cells treated with MeOH or SSA. Graph shows SSA sample values expressed as a fold increase over MeOH values (given a value of 1) after adjustment to U1 RNA levels. (**E**) Quantitative 3′ RACE analysis of β-globin RNA from samples isolated from MeOH or SSA treated βWT, βWTCoTC and βtSA HEK cells. Diagram shows tagged reverse transcription and intron 2 primers. For each cell line. SSA samples are expressed as a fold change compared with MeOH samples (given a value of 1, following normalization to U1 levels). Error bars show SD from at least three biological repeats. Asterisk denotes *P* < 0.05 for SSA-dependent changes.

We first determined the effect of SSA on β-globin splicing using both the βWT and βtSA HEK cell lines. Total RNA was isolated from cells treated with SSA or its methanol (MeOH) solvent. Treatment was limited to 3 h to limit potential off-target effects of splicing inhibition. Real-time PCR was then used to quantitate the level of unspliced intron 2 (Ex2-In2), intron 2 lariat, spliced (Ex2-Ex3) and total (Ex3) β-globin transcripts (Figure 3A and B). In βWT cells, SSA increased the level of intron 2 and reduced the amount of lariat intermediate, demonstrating a strong inhibition of splicing before catalysis. By this measure, inhibition was more efficient than with the U1 ASO. Intron 2 levels were also increased in βtSA cells treated with SSA owing to inhibition of a less frequent cryptic splicing event (Supplementary Figure S3). Little SSA-dependent change in total or spliced βWT RNA was seen potentially because stable processed transcripts are present from before this short splicing block. As expected, total and correctly spliced βtSA transcripts were present at low levels or absent, respectively.

To analyse 3′ end formation, we detected β-globin transcripts that were not cleaved at the pA site (Figure 3C). We also analysed 3′ flanking RNA, as transcriptional read-through is a feature of improper pA site function. SSA treatment resulted in a modest increase in uncleaved βWT pA sites (∼1.6-fold) and 3′ flanking (∼2-fold) transcripts indicating a potential effect on 3′ end formation. However, an SSA-dependent effect of similar magnitude was observed in βtSA samples. Given that pA cleavage is substantially less efficient for βtSA (Figure 1C), it is unlikely that a 3′ end processing defect is responsible for this.

To further determine the effect of SSA on β-globin 3′ end formation, we used another cell line (βWTCoTC). This is the same as βWT but has a sequence (the CoTC element) in the 3′ flank, which promotes transcriptional termination in both plasmid and chromosomal environments (30,34). An efficient termination process would reduce the synthesis of any read-through RNA, which might be somehow stabilized following SSA treatment. More importantly, however, if the effect of SSA is on processing at the β-globin pA site, this would still be seen, as this sequence is identical to βWT. Figure 3D shows that SSA inhibits terminal intron splicing on βWTCoTC RNA as for βWT, as intron 2 and lariat signals are, respectively, increased and decreased by similar amounts. However, in βWTCoTC cells, SSA did not affect the level of non-pA cleaved or flank RNA. This result does not support substantial inhibition of β-globin 3′ end formation by SSA. In line with this conclusion, an independent study showed a modest reduction in β-globin read-though, following 24 h of SSA treatment, using a similar stable integrate (48).

If 3′ end formation does continue in the presence of SSA, cleaved and polyadenylated transcripts that are also unspliced will accumulate. To test this and any function for a tSA site, we used our 3′ RACE approach to detect these transcripts (Figure 3E). SSA induced a 2-fold increase in unspliced 3′ end processed RNA recovered from βWT and βWTCoTC cells. Importantly, however, there was a lack of SSA effect on 3′ end formation of βtSA transcripts, which is in contrast to what is seen by U1 inhibition. Thus, a tSA site is required for 3′ end formation when splicing is impaired by SSA, but not when prior steps in spliceosome assembly are blocked by inhibiting U1. This is consistent with the idea that recruitment of U1 inhibits cleavage and polyadenylation until a functional tSA site is encountered. However, although a tSA site is required for 3′ end formation in the presence of SSA, subsequent intron removal is less critical, as unspliced 3′ end processed βWT and βWTCoTC RNA accumulates.

### U1 snRNA inhibition and SSA do not affect 3′ end formation of P27 and Myc transcripts

**Figure 4.**
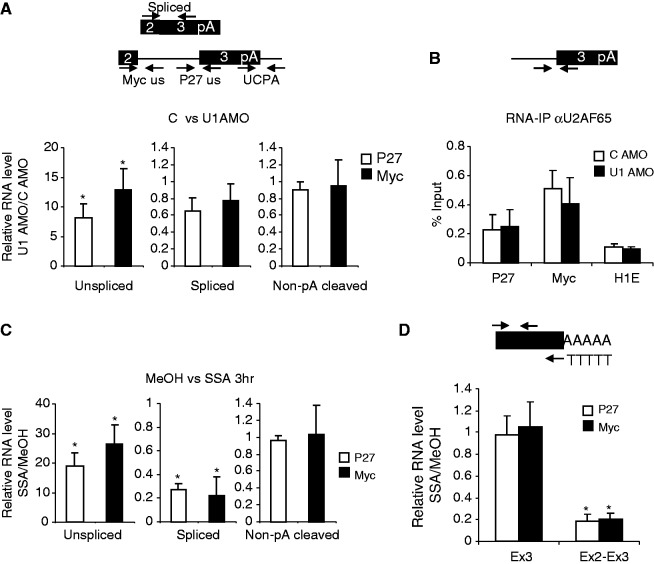
(**A**) Diagram shows representative penultimate and terminal exons from P27 and Myc genes with primers used to analyse unspliced Myc (Myc US), unspliced P27 (P27 US), non-pA cleaved (UCPA) and spliced RNAs. Graphs show real-time PCR analyses of these species in cells treated with control or U1 AMO. Values are from U1 AMO samples and expressed as fold change relative to those obtained in control-treated cells (set to 1 after normalizing to U1 levels). (**B**) RIP analysis of U2AF65 binding to the tSA site of Myc and P27 pre-mRNA in control or U1 AMO-treated HeLa cells. Quantitation is expressed as percentage input. Values obtained from Histone H1E (used as a negative control) are also shown. (**C**) Real-time PCR analysis of unspliced, spliced and non-pA cleaved Myc and P27 transcripts in cells treated with MeOH or SSA. Values are from SSA samples and expressed as fold change relative to those obtained in control-treated cells (set to 1 after normalizing to U1 levels). (**D**) Real-time PCR analysis of total (Ex3) and spliced (Ex2-Ex3) 3′ end-processed Myc and P27 transcripts in cells treated with MeOH or SSA. Diagram shows the primer designed to detect polyadenylated RNA. Values are from SSA samples and expressed as fold change relative to those obtained in MeOH-treated cells following normalization to U1 levels. Error bars show SD from at least three biological repeats. Asterisk denotes *P* < 0.05 for differences following splicing inhibition.

We next wanted to analyse the effects of U1 inhibition and SSA on transcripts synthesised from genes in their natural chromosomal context. P27 and Myc transcripts were chosen, as they have previously been used to study effects of SSA (48,49). Moreover, they have no obvious sites for premature cleavage and polyadenylation, which is a widespread consequence of U1 inhibition (22). We first tested the effects of U1 inhibition by electroporating HeLa cells with a control AMO or one that binds the pre-mRNA interacting region of U1 (22). An AMO was used, as it can be electroporated into HeLa cells facilitating a more rapid inhibition of splicing than ASO transfection (we were unable to introduce AMOs into our HEK cells). Accordingly, total RNA was isolated after 3 h and reverse transcribed. We then used primer sets to detect unspliced, spliced and non-pA cleaved P27 or Myc RNA (Figure 4A). Consistent with splicing inhibition, we observed a substantial increase in unspliced RNA for both transcripts, with a more modest decrease in spliced RNA, likely owing to its stability. There was no change in the level of non-pA cleaved RNA indicating that 3′ end processing remains functional in the absence of U1 and early spliceosome assembly. Finally, U2AF65 RIP analysis revealed that it associates with P27 and Myc transcripts with similar efficiency in control and U1 AMO-treated cells (Figure 4B). Thus, although U1 AMO inhibits U1 binding and pre-mRNA splicing, the intact 3′ splice site can still recruit some U2AF. This might be due to terminal exon definition requiring an intact 3′ splice site and pA signal (11,50), which are present in this situation. In sum, these data show that 3′ end processing can proceed when splicing is impaired by preventing U1 from interacting with pre-mRNA. This is similar to our β-globin observations and reports that many pA signals are activated when U1 is inhibited in this way (21,22).

We next analysed the effects of blocking later steps in spliceosome assembly using SSA. Total RNA was isolated from cells treated with MeOH or SSA. Following reverse transcription, cDNA was analysed with primers to detect unspliced, non-pA cleaved and spliced RNA from Myc and P27 genes (Figure 4C). In both cases, SSA treatment caused a large accumulation of unspliced transcripts when compared with the MeOH control. SSA also caused a strong reduction in the level of spliced Myc and P27 RNA consistent with a substantial inhibition of splicing. Despite this, there was little effect of SSA on the level of non-pA cleaved RNA arguing that, although splicing was inhibited, 3′ end formation was still occurring. Consistently, an anchored oligo-dT RT-PCR revealed little effect of SSA on the total level of 3′ end processed P27 and Myc RNA, despite the spliced versions of each being depleted (Figure 4D). These effects were also observed when lower concentrations of SSA were used and when shorter time points were used (Supplementary Figure S4). This is again consistent with our β-globin analyses.

### A U4 AMO is a rapid and potent splicing inhibitor

**Figure 5.**
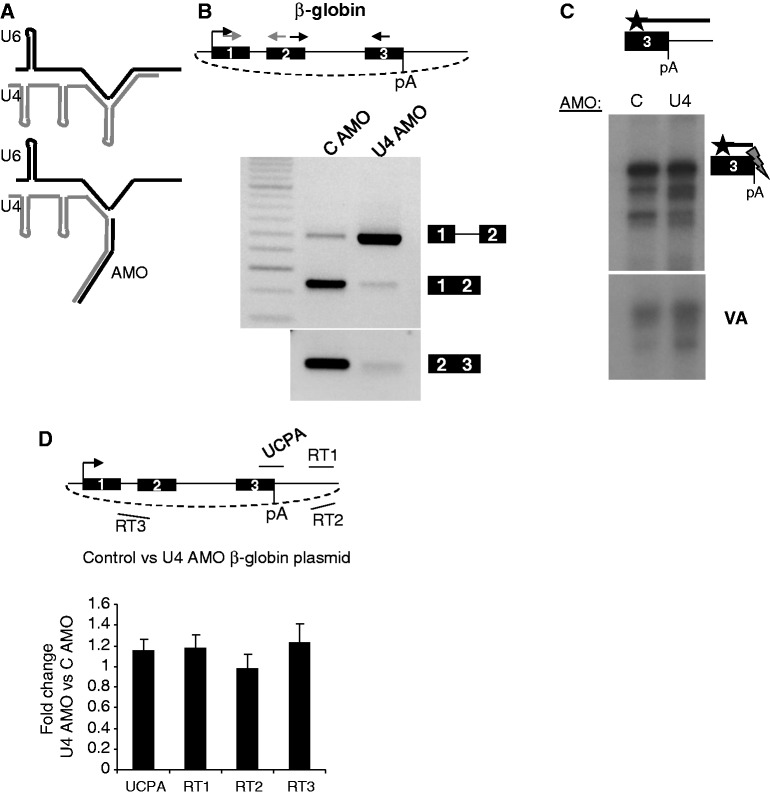
(**A**) Diagram depicting target of the U4 AMO. U4 (grey) and U6 (black) are extensively base paired, but this is predicted to be disrupted by the U4 AMO. (**B**) Analysis of the effect of U4 AMO treatment of β-globin splicing. The diagram shows β-globin expression plasmid and the position of primers used for real-time PCR (Ex1-Ex2; grey arrows Ex2-Ex3; black arrows). Top gel shows PCR products of amplification with Ex1-Ex2 primers with bands corresponding to spliced and unspliced products indicated. Lower gel shows the result of PCR with Ex2-Ex3 primers. (**C**) S1 Nuclease analysis of pA site cleaved β-globin RNAs in samples from control and U4 AMO-treated cells. The diagram shows 3′ end of β-globin gene and end-labelled probe complementary to pA site region (black line with star). The gel shows products following S1 nuclease digestion with pA cleaved products and those deriving from co-transfected VA indicated. (**D**) Analysis of non-pA cleaved (UCPA) and flanking region transcripts in RNA isolated from control and U4 AMO-treated HeLa cells transfected with β-globin plasmid. Diagram shows the plasmid and amplicons detected. Graph shows quantitation following normalization to co-transfected VA RNA. Values are expressed as a fold change compared with control AMO-treated samples, which are given a value of 1. Error bars show SD from at least three biological repeats.

We found it surprising that SSA-induced splicing inhibition did not observably impact on 3′ end formation. To be more confident about this finding, we sought an alternative means of inhibiting a similar step of splicing and used an AMO directed to U4 (Figure 5A). The region of U4 targeted by our AMO is required to form a stem with U6 that is essential for active splicing (51). Pre-mRNA injected into *Xenopus* Oocytes that contain U4 lacking this region can only associate with U1 and U2 (52). Consistently, treatment with our U4 AMO causes a substantial reduction in the level of chromatin-associated U4, U5 and U6 but has less effect on the recovery of U1 and U2 (Supplementary Figure S5).

We validated the effectiveness of the U4 AMO *in vivo* by electroporating it into HeLa cells that were transiently transfected with a β-globin expression plasmid (Figure 5B). Total RNA was isolated after 3 h to avoid off target effects, and cDNA was generated with random hexamers. PCR was performed with primers in β-globin exons 1 and 2 or in exons 2 and 3. In control AMO samples, these primers detected mostly spliced RNA with a fainter band corresponding to unspliced transcripts. In the U4 AMO sample, we observed a reduction in spliced RNA and a large increase in unspliced precursor. Splicing is inhibited before catalysis because accumulation of lariat from intron 2 was strongly reduced by the U4 AMO (Supplementary Figure S6A).

We next tested whether the reduced β-globin splicing caused corresponding effects on 3′ end formation. We performed S1 nuclease analysis on the same RNA samples as Figure 5B (Figure 5C). Transcripts were probed with end-labelled DNA specific to the β-globin 3′ end or the transfection control VA RNA. Levels of VA RNA are similar in both samples indicating equivalent transfection. Strikingly, there was also little difference in the level of RNA cleaved at the pA site. Thus, although splicing is severely compromised by U4 inhibition, 3′ end processing still occurs. As with SSA, unspliced 3′ end processed RNA was produced following U4 inhibition in a manner requiring a tSA site (Supplementary Figure S6B and C). Analysis of prematurely processed NR3C1 revealed that the ability of U1 to bind pre-mRNA and inhibit premature 3′ end formation is largely unaffected by U4 AMO treatment (Supplementary Figure S7).

It is well established that there is an intimate relationship between pA signal function and transcriptional termination. Therefore, as a final measure of the effect of U4 inhibition on 3′ end formation, we checked its effect on transcriptional termination (Figure 5D). RNA was isolated from transfected control or U4 AMO-treated cells, and qRT-PCR was performed to detect non-pA cleaved transcripts and read-through RNA from three different positions 3′ of the pA signal. Signals were normalized following adjustment to co-transfected VA RNA levels. There was no increase in read-through RNA at any of the positions tested, suggesting that termination continues to occur on the β-globin plasmid following U4 AMO treatment. Termination is therefore independent of intron removal when splicing is blocked through inhibition of U4.

### U4 AMO inhibits P27 and Myc splicing, but not 3′ end formation

**Figure 6.**
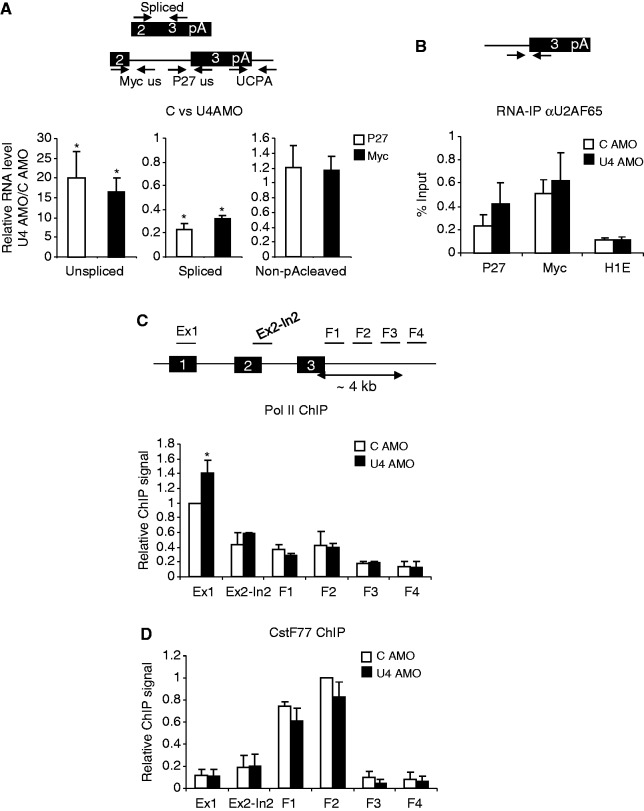
(**A**) Real-time PCR analysis of unspliced, spliced and non-pA cleaved Myc and P27 transcripts in cells treated with control or U4 AMOs. Values are from U4 AMO samples and expressed as fold change relative to those obtained in control AMO-treated cells (set to 1) following normalization to U1 levels. (**B**) RIP analysis of U2AF65 binding to the tSA site of Myc or P27 pre-mRNA in control or U4 AMO-treated HeLa cells. Quantitation is expressed as percentage input. Values obtained from Histone H1E (used as a negative control) are also shown. (**C**) ChIP analysis of Pol II loading on P27 and Myc genes in cells treated with control or U4 AMO. Diagrams show each gene with approximate positions of ChIP primer sets indicated. Graph shows quantitation where, following normalization to the TAF7 gene where ChIP signal is expressed as a fold change in comparison with the +150 region in control cells. (**D**) ChIP analysis of CstF77 loading on P27 and Myc genes in cells treated with control or U4 AMO. Graph shows quantitation where, following normalization to the TAF7 gene where ChIP signal is expressed as a fold change in comparison with the flank 2 region value in control cells. Error bars show SD from at least three biological repeats. Asterisk denotes where differences between control and U4 AMO-treated samples are *P* < 0.05.

We next analysed the effect of U4 inhibition on endogenous P27 and Myc transcripts by performing qRT-PCR to detect unspliced, spliced and non-pA cleaved sampled in control or U4 AMO-treated cells (Figure 6A). We observed a large increase in unspliced RNA levels with a reduction of spliced RNA on U4 AMO treatment demonstrating effective splicing inhibition. Consistent with a lack of effect on 3′ end formation, U4 inhibition did not increase the amount of non-pA cleaved RNA that was recovered. As with SSA, this result was maintained at lower concentrations of AMO, which makes it unlikely that saturation of a key factor is responsible for the observation (Supplementary Figure S8). A lack of effect on processing at the P27 and Myc pA sites was also confirmed using an anchored oligo-dT reverse transcription and real-time PCR approach (Supplementary Figure S9).

We also performed U2AF65 RIP to assess its binding to the tSA site of Myc and P27 transcripts in control and U4 AMO samples (Figure 6B). A similar proportion of tSA containing transcripts were bound by U2AF65 in control and U4 AMO samples. However, unlike with U1 AMO, the association of U1 snRNA with pre-mRNA remains efficient under these conditions (Supplementary Figure S7). This is consistent with the prediction, from previous data and from the classical splicing pathway (52), that inhibition of U4 in this way blocks splicing following the recruitment of U1 and U2 to the 5′ and 3′ splice sites.

### U4 AMO does not affect recruitment of Pol II and CstF77 to the Myc gene

Having analysed the pre-mRNA levels and 3′ splice site occupancy by U2AF65, we next tested the effects of U4 inhibition on Pol II density across the Myc gene. This is important as changes in the level of various pre-mRNA species may also result from differences in the abundance of transcribing Pol II. Indeed, splicing perturbation is sometimes associated with changes in Pol II loading on genes (35,36,53). We performed Pol II ChIP (using the N20 antibody) in control and U4 AMO-treated cells using primer pairs along the Myc gene. Primer sets were also located within the 3′ flanking region to analyse transcriptional termination (Figure 6C). Comparing control and U4 AMO samples revealed no difference in Pol II loading over any of the positions tested including those downstream of the pA site. This shows that U4 inhibition does not cause changes in transcription or defects in termination as measured by ChIP.

We next wanted to test the effects of U4 inhibition on the recruitment of cleavage and polyadenylation factors to the Myc gene. For many such factors, this is readily observed by ChIP. For instance, members of the CstF complex are found recruited at and beyond the pA signal consistent with their biological function in 3′ end formation (54). We therefore assayed the recruitment of CstF77 in both control and U4 AMO-treated samples using ChIP (Figure 6D). In control cells, CstF77 signal was strongest at positions downstream of the pA site, which is consistent with previous observations (54). Importantly, a similar profile was observed in U4 AMO-treated samples indicating that, even when splicing is impaired in this manner, the 3′ end processing complex is present to potentially execute rapid 3′ end formation.

### U4 AMO causes release of pre-mRNA into the nucleoplasm and enhances its stability

**Figure 7.**
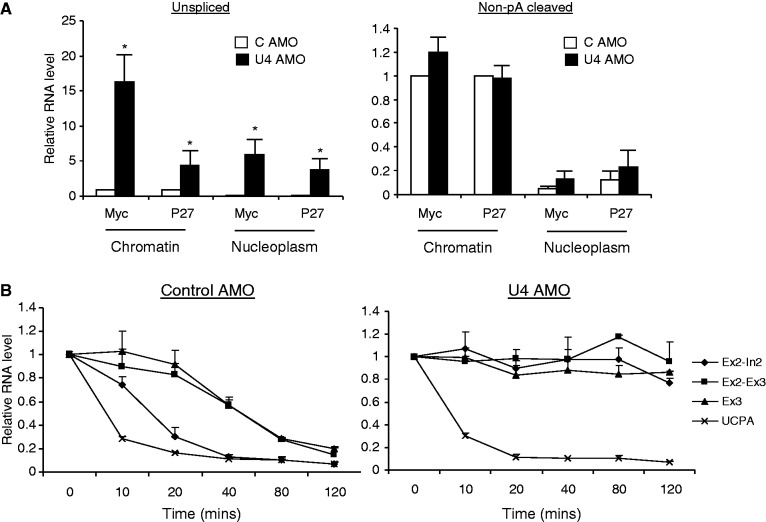
(**A**) Analysis of unspliced (left graph) and non-pA cleaved (right graph) P27 and Myc RNA in chromatin and nucleoplasm of HeLa cells treated with control or U4 AMO. Graphs show quantitation as a fold change following normalization to U1 levels. Values are expressed relative to those obtained in chromatin samples from control AMO cells, which are given a value of 1. (**B**) Analysis of Myc transcript decay in control (left graph) and U4 AMO (right graph)-treated cells subsequently treated with ActD. Graphs show relative level of unspliced intron 2 (Ex2-In2), spliced (Ex2-Ex3), total (Ex3) and non-pA cleaved (UCPA) transcripts at each time point with values displayed relative to the amount present at t0, which is given a value of 1. Error bars show SD from at least three biological repeats. Asterisk denotes where differences between control and U4 AMO-treated samples are *P* < 0.05.

Mutations that prevent pre-mRNA splicing cause aberrant transcripts to be retained at their site of synthesis (35,36,53,55,56). However, it is notable that cis-mutations block splicing at earlier points to where the U4 AMO is predicted to act. With this in mind, we assessed the intra-nuclear distribution of unspliced pre-mRNA following control or U4 AMO treatment. A well-established technique was used to isolate chromatin-associated and nucleoplasmic RNA (33). We then used qRT-PCR to detect unspliced and non-pA cleaved P27 and Myc transcripts (Figure 7A). U4 AMO treatment caused an increase in the level of chromatin-associated unspliced P27 and Myc transcripts consistent with inhibition of co-transcriptional splicing. Strikingly, U4 AMO treatment also caused a strong accumulation of unspliced transcripts in the nucleoplasm, indicating that they are released from chromatin. In contrast, non-pA cleaved RNA was largely confined to the chromatin fraction in control and U4 AMO samples consistent with co-transcriptional 3′ end formation in both conditions. This experiment suggests that, in contrast to RNA containing *cis*-mutations that inactivate early steps in splicing, the U4 AMO results in the accumulation of many unspliced transcripts in the nucleoplasm. This is in line with the independent observation that SSA induces the accumulation of polyadenylated RNA in nucleoplasmic foci (45).

Aberrant pre-mRNAs are often rapidly degraded as a consequence of their retention at transcription sites (35). We therefore wished to test the hypothesis that the 3′ end processing and release of pre-mRNAs into the nucleoplasm might stabilize them instead. To do so, we treated cells with control and U4 AMOs before supplementing media with actinomycin D (ActD) to inhibit transcription. The levels of unspliced, spliced, total and non-pA cleaved transcripts were then measured at various time points thereafter using RT-PCR on total RNA. Figure 7B shows the results obtained for the Myc transcripts in this experiment. In control AMO samples, total and spliced transcripts are relatively stable up until 40 min, and then they decay. In contrast, both unspliced and non-pA cleaved transcripts begin to disappear after only 10 min, as a consequence of their rapid processing or degradation. In U4 AMO-treated samples, unspliced, spliced and total RNAs were highly stable. However, the non-pA cleaved transcripts behaved identically to those in control samples. These data provide further support for our model that U4 inhibition does not impair pA site cleavage.

## DISCUSSION

Our findings are consistent with the following model for the interconnections between splicing and 3′ end processing. Assembly of U1 on the 5′ splice site of the terminal intron occurs first and serves to inhibit cleavage and polyadenylation. To overcome this inhibition, a tSA site is required to promote terminal exon definition in combination with a pA signal. Our data indicate that these early interactions are vital to couple splicing and 3′ end formation, as a tSA site mutation inhibits both processes. Although splicing catalysis and intron excision occur shortly after terminal exon definition is established under normal circumstances, our results strongly suggest that splicing catalysis is dispensable for coupled 3′ end formation. This is shown by the apparently efficient 3′ end formation that takes place in the presence of SSA or U4 AMO, both of which block splicing before any catalytic steps.

Our data indicate that 3′ end formation is linked to splicing once U1 is recruited to the transcript because, once this occurs, cleavage and polyadenylation is inhibited unless a functional 3′ splice site is encountered. Such a mechanism is consistent with observations of widespread 3′ end formation within introns on inhibition of U1 (17,21,22,57). This function of U1 must somehow be negated at gene ends. Although removal of the intron is one way that this could happen, our data suggest that 3′ end formation can still occur when splicing catalysis is blocked by SSA or U4 AMO. Under these conditions, U1 is still able to inhibit pA site processing, suggesting that it remains bound to pre-mRNA [(22) and Supplementary Figure S7]. Therefore, the tSA site must play a role in buffering its negative effects or promoting cleavage and polyadenylation. This would presumably involve the recruitment of factors with this function.

U2AF65 is involved in 3′ splice site recognition, terminal exon definition and has been shown to stimulate cleavage and polyadenylation. Interestingly, U2AF65 can promote β-globin 3′ end processing when tethered artificially to the transcript, suggesting that this function does not require removal of an intron (40). Our finding that reduced 3′ end formation on tSA mutation is also associated with a lack of U2AF65 recruitment is correlative with a potential positive function in 3′ end formation. Other factors involved at the 3′ splice site may also play a role here. In particular, a previous study identified SF3b components as being important for coupling splicing to 3′ end formation *in vitro* (15). SF3b is also needed for branch-point recognition by U2, disruption of which shows a mild 3′ end processing defect for some transcripts (Supplementary Figure S10). More generally, most splicing factors described as having potential function in cleavage and polyadenylation are associated with either U1 or U2 recruitment (15,40,58). This is consistent with our model that early events in splicing are important for coupling.

Global analyses of nascent transcripts show co-transcriptional removal of a large number of introns (24,26). However, co-transcriptional splicing is reported as being less efficient for introns located nearer to the end of pre-mRNAs (25,28). In those instances, 3′ end formation might occur more rapidly than splicing. Indeed, available analyses indicate that some pA sites are processed in a matter of seconds with splicing occurring in minutes (59,60). Similarly, our actinomycin time course indicates that unspliced Myc transcripts are processed more slowly than non-pA cleaved RNA (Figure 7B). Our data indicate that splicing and 3′ end formation can be coupled in these situations owing to recruitment of U1 to the 5′ splice site and U2AF65 (and presumably U2) to the 3′ splice site. This is likely to be a rapid process, as both factors are present on transcribing Pol II (41,61,62).

It is well documented that many splice defective transcripts in lower and higher eukaryotes are rapidly degraded (36,53,63). Although this is also true of some transcripts following SSA treatment, other pre-mRNAs accumulate and are stabilized (35). We observe a similar accumulation and stabilization of P27 and Myc transcripts following U4 AMO treatment. This correlates with their release into the nucleoplasm, which we propose is facilitated by the continued cleavage and polyadenylation that occurs under these splice inhibitory conditions. Consistently, immunofluorescence experiments demonstrated that SSA or U4 treatment causes a large accumulation of polyadenylated RNA in nucleoplasmic foci that co-localize with the splicing speckle marker, SC35 (45) and our unpublished results. Previous work showed that unspliced P27 pre-mRNA can be translated following SSA treatment (45), which may implicate the nonsense-mediated decay pathway in the turnover of these and similar transcripts. However, it is not known how unspliced pre-mRNA that accumulates in intra-nuclear speckles is eventually exported to the cytoplasm.

We have shown that the tight co-transcriptional coupling between splicing and 3′ end formation is established early during terminal exon definition following U1 recruitment to the 5′ splice site and recognition of the tSA site. These early events seem sufficient to permit 3′ end formation, which continues following blocks to later steps in the splicing process.

## SUPPLEMENTARY DATA

Supplementary Data are available at NAR Online: Supplementary Table 1, Supplementary Figures 1–10 and Supplementary Methods.

## FUNDING

Research career development award from the Wellcome Trust [088499/Z/09/2] and Centre core grant from the Wellcome Trust [092076]. Funding for open access charge: The Wellcome Trust.

*Conflict of interest statement*. None declared.

## Supplementary Material

Supplementary Data
