# Supplementary material for: Splicing-coupled 3′ end formation requires a terminal splice acceptor site, but not intron excision
Source: Nucleic Acids Res. 2013 May 28;41(14):7101–14. doi: 10.1093/nar/gkt446 (PMC3737548; doi:10.1093/nar/gkt446)
Supplement: Supplementary Data [file supp_41_14_7101__index.html]

Splicing-coupled 3′ end formation requires a terminal splice acceptor site, but not intron excision — Splicing-coupled 3′ end formation requires a terminal splice acceptor site, but not intron excision — Supplementary Data 

# Splicing-coupled 3′ end formation requires a terminal splice acceptor site, but not intron excision

## Supplementary Data

files

**Files in this Data Supplement:**

- Supplementary Data - ppt file
- Supplementary Data - doc file
