# Supplementary material for: Splicing-coupled 3′ end formation requires a terminal splice acceptor site, but not intron excision
Source: Nucleic Acids Res. 2013 May 28;41(14):7101–14. doi: 10.1093/nar/gkt446 (PMC3737548; doi:10.1093/nar/gkt446)
Supplement: Supplementary Data [file supp_gkt446_nar-00182-a-2013-File003.ppt]

## Slide 1
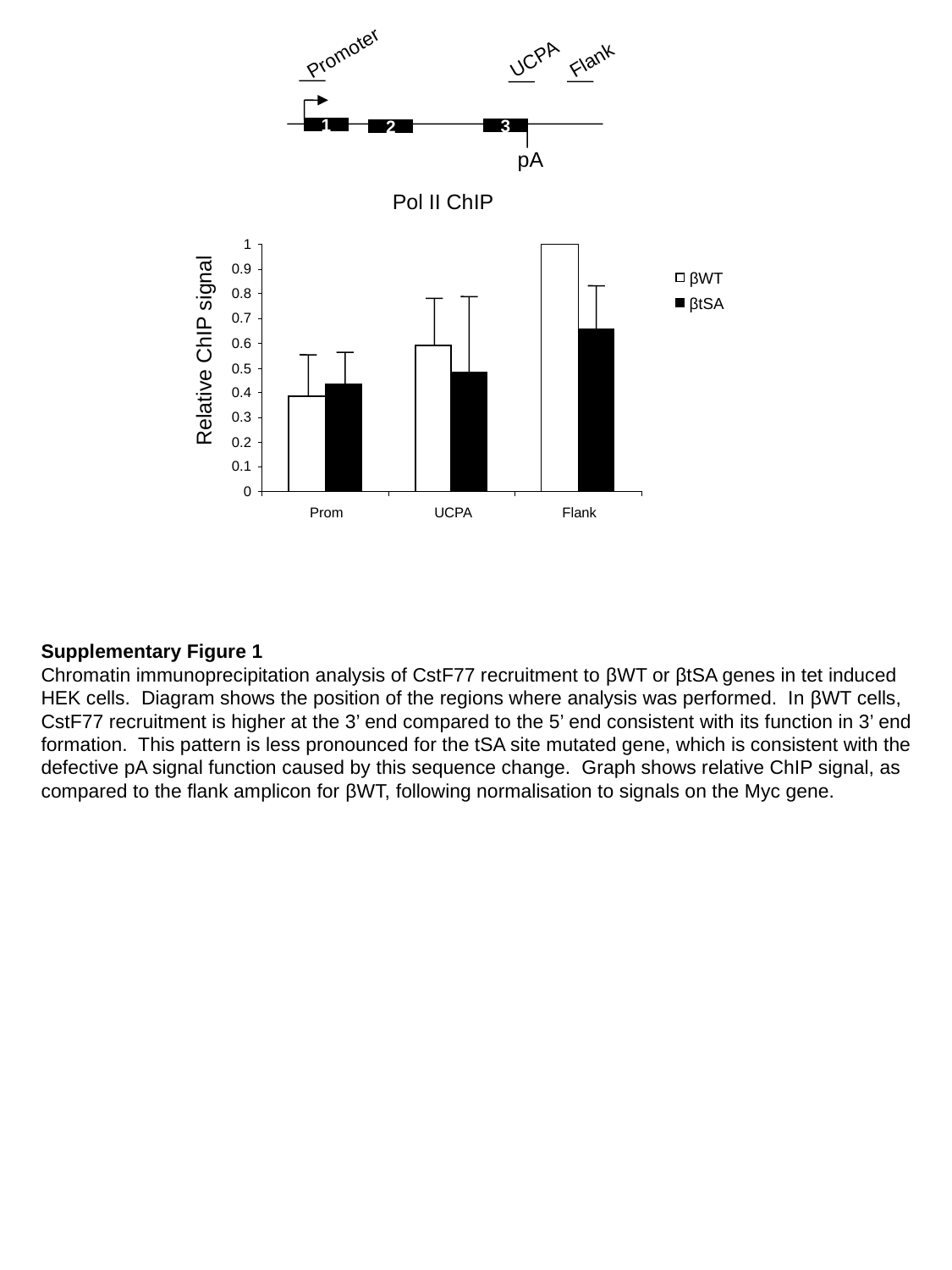

Promoter
UCPA
Flank
1
3
2
pA
Pol II ChIP
1
0.9
βWT
0.8
βtSA
0.7
Relative ChIP signal
0.6
0.5
0.4
0.3
0.2
0.1
0
Prom
UCPA
Flank
Supplementary Figure 1
Chromatin immunoprecipitation analysis of CstF77 recruitment to βWT or βtSA genes in tet induced HEK cells. Diagram shows the position of the regions where analysis was performed. In βWT cells, CstF77 recruitment is higher at the 3’ end compared to the 5’ end consistent with its function in 3’ end formation. This pattern is less pronounced for the tSA site mutated gene, which is consistent with the defective pA signal function caused by this sequence change. Graph shows relative ChIP signal, as compared to the flank amplicon for βWT, following normalisation to signals on the Myc gene.

## Slide 2
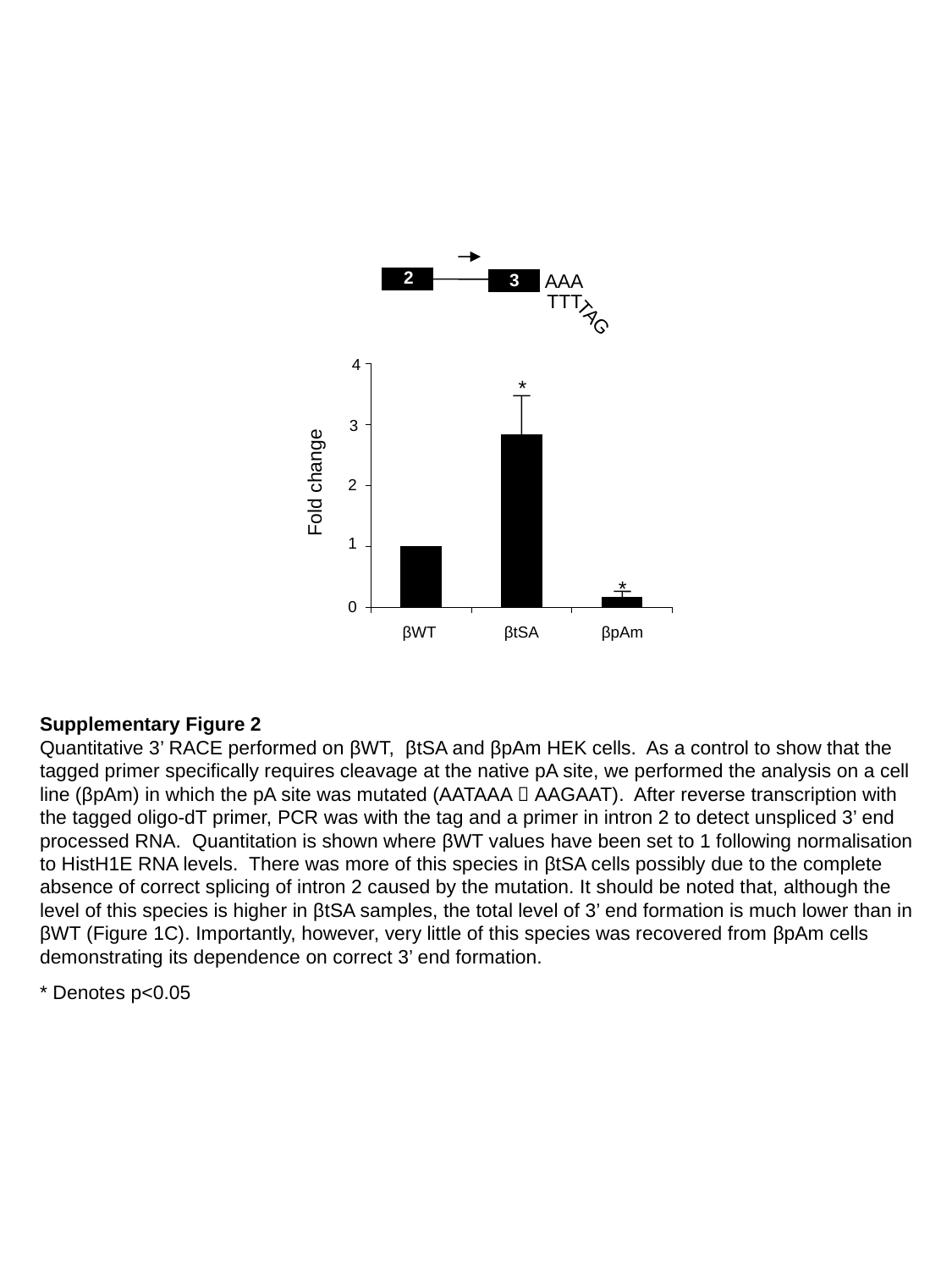

3
AAA
2
TTT
TAG
4
*
3
Fold change
2
1
*
0
βWT
βtSA
βpAm
Supplementary Figure 2
Quantitative 3’ RACE performed on βWT, βtSA and βpAm HEK cells. As a control to show that the tagged primer specifically requires cleavage at the native pA site, we performed the analysis on a cell line (βpAm) in which the pA site was mutated (AATAAA  AAGAAT). After reverse transcription with the tagged oligo-dT primer, PCR was with the tag and a primer in intron 2 to detect unspliced 3’ end processed RNA. Quantitation is shown where βWT values have been set to 1 following normalisation to HistH1E RNA levels. There was more of this species in βtSA cells possibly due to the complete absence of correct splicing of intron 2 caused by the mutation. It should be noted that, although the level of this species is higher in βtSA samples, the total level of 3’ end formation is much lower than in βWT (Figure 1C). Importantly, however, very little of this species was recovered from βpAm cells demonstrating its dependence on correct 3’ end formation.
* Denotes p<0.05

## Slide 3
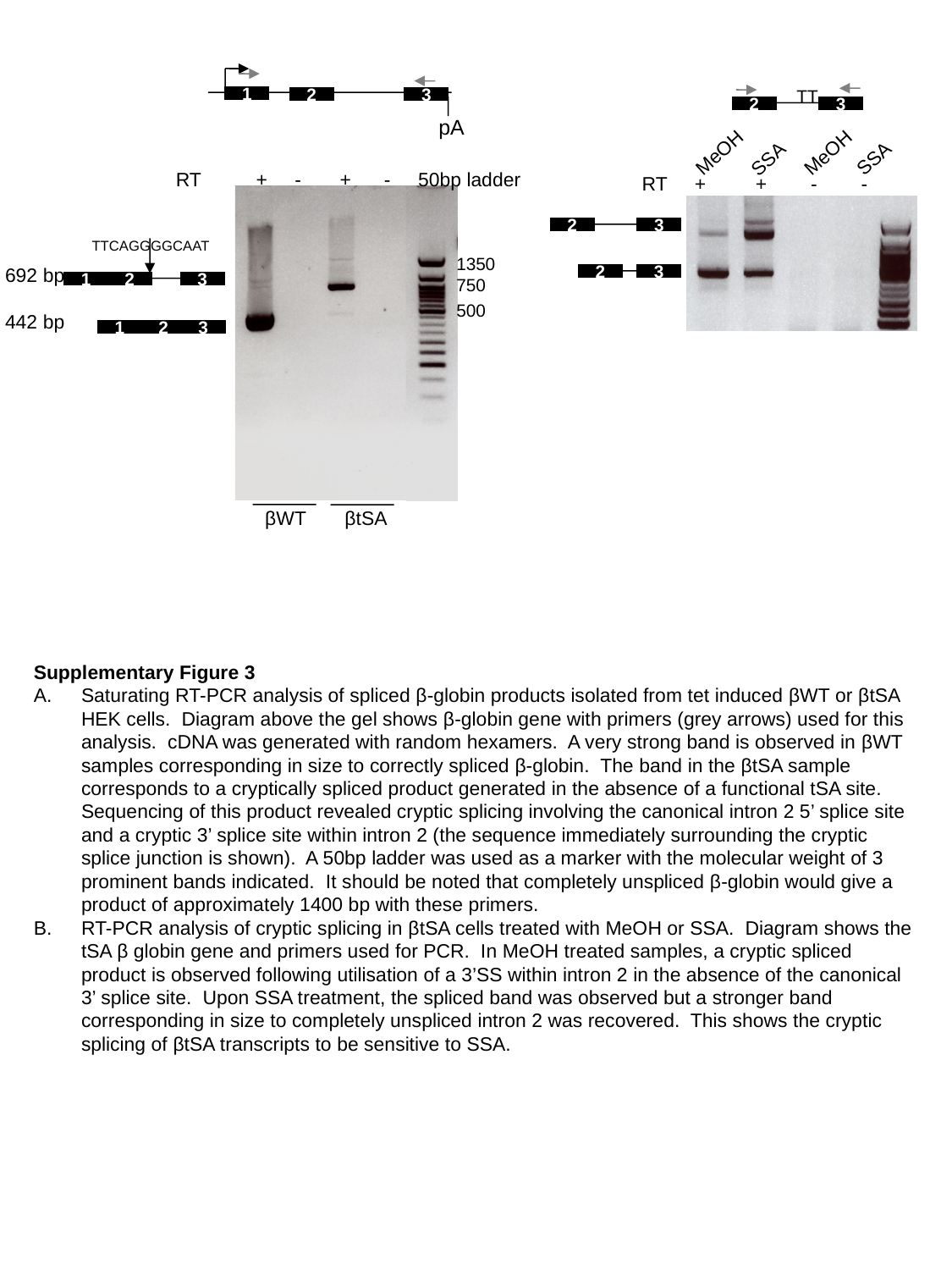

TT
1
3
2
2
3
pA
MeOH
MeOH
SSA
SSA
RT + - + - 50bp ladder
RT + + - -
2
3
TTCAGGGGCAAT
1350
750
500
692 bp
442 bp
2
3
1
2
3
1
2
3
βWT βtSA
Supplementary Figure 3
Saturating RT-PCR analysis of spliced β-globin products isolated from tet induced βWT or βtSA HEK cells. Diagram above the gel shows β-globin gene with primers (grey arrows) used for this analysis. cDNA was generated with random hexamers. A very strong band is observed in βWT samples corresponding in size to correctly spliced β-globin. The band in the βtSA sample corresponds to a cryptically spliced product generated in the absence of a functional tSA site. Sequencing of this product revealed cryptic splicing involving the canonical intron 2 5’ splice site and a cryptic 3’ splice site within intron 2 (the sequence immediately surrounding the cryptic splice junction is shown). A 50bp ladder was used as a marker with the molecular weight of 3 prominent bands indicated. It should be noted that completely unspliced β-globin would give a product of approximately 1400 bp with these primers.
B.	RT-PCR analysis of cryptic splicing in βtSA cells treated with MeOH or SSA. Diagram shows the tSA β globin gene and primers used for PCR. In MeOH treated samples, a cryptic spliced product is observed following utilisation of a 3’SS within intron 2 in the absence of the canonical 3’ splice site. Upon SSA treatment, the spliced band was observed but a stronger band corresponding in size to completely unspliced intron 2 was recovered. This shows the cryptic splicing of βtSA transcripts to be sensitive to SSA.

## Slide 4
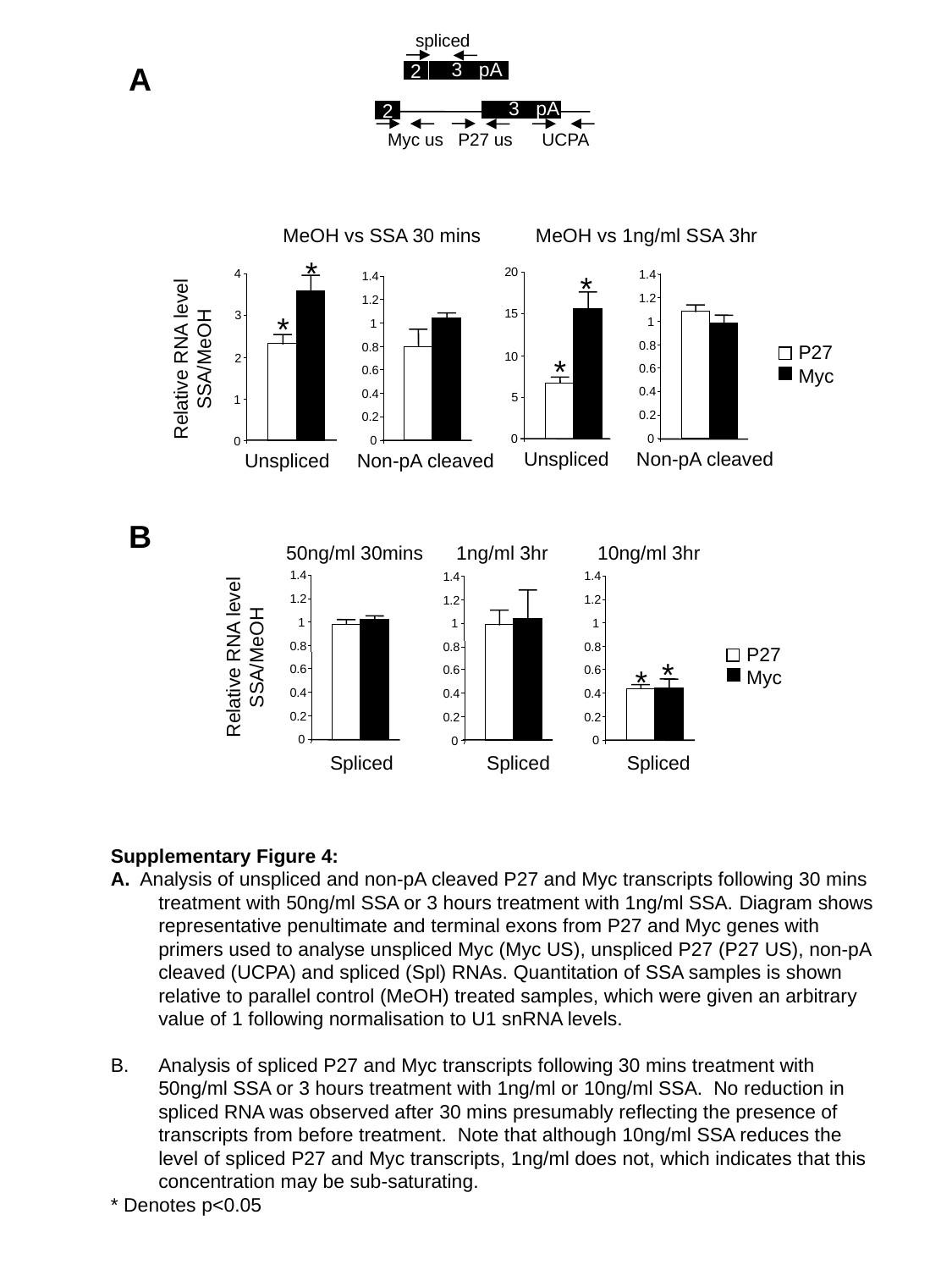

spliced
3 pA
A
B
2
3 pA
2
Myc us P27 us UCPA
MeOH vs SSA 30 mins MeOH vs 1ng/ml SSA 3hr
*
*
20
4
1.4
1.4
1.2
1.2
*
15
3
1
1
Relative RNA level
SSA/MeOH
P27
Myc
0.8
0.8
*
10
2
0.6
0.6
0.4
0.4
5
1
0.2
0.2
0
0
0
0
Unspliced Non-pA cleaved
Unspliced Non-pA cleaved
50ng/ml 30mins 1ng/ml 3hr 10ng/ml 3hr
1.4
1.4
1.4
1.2
1.2
1.2
1
1
1
Relative RNA level
SSA/MeOH
P27
Myc
0.8
0.8
0.8
*
*
0.6
0.6
0.6
0.4
0.4
0.4
0.2
0.2
0.2
0
0
0
Spliced Spliced Spliced
Supplementary Figure 4:
A. Analysis of unspliced and non-pA cleaved P27 and Myc transcripts following 30 mins treatment with 50ng/ml SSA or 3 hours treatment with 1ng/ml SSA. Diagram shows representative penultimate and terminal exons from P27 and Myc genes with primers used to analyse unspliced Myc (Myc US), unspliced P27 (P27 US), non-pA cleaved (UCPA) and spliced (Spl) RNAs. Quantitation of SSA samples is shown relative to parallel control (MeOH) treated samples, which were given an arbitrary value of 1 following normalisation to U1 snRNA levels.
Analysis of spliced P27 and Myc transcripts following 30 mins treatment with 50ng/ml SSA or 3 hours treatment with 1ng/ml or 10ng/ml SSA. No reduction in spliced RNA was observed after 30 mins presumably reflecting the presence of transcripts from before treatment. Note that although 10ng/ml SSA reduces the level of spliced P27 and Myc transcripts, 1ng/ml does not, which indicates that this concentration may be sub-saturating.
* Denotes p<0.05

## Slide 5
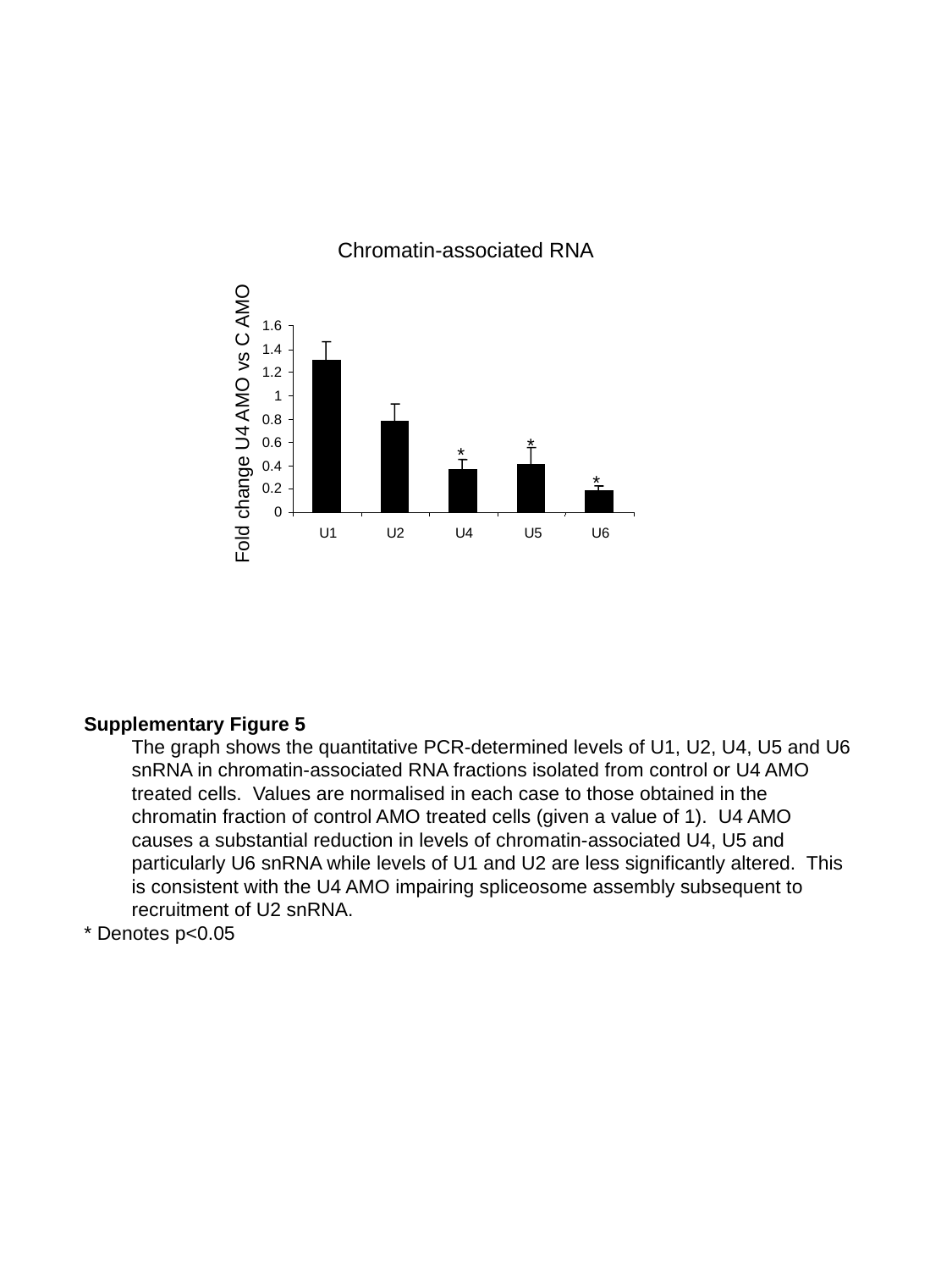

Chromatin-associated RNA
1.6
1.4
1.2
1
Fold change U4 AMO vs C AMO
0.8
*
0.6
*
0.4
*
0.2
0
U1
U2
U4
U5
U6
Supplementary Figure 5
	The graph shows the quantitative PCR-determined levels of U1, U2, U4, U5 and U6 snRNA in chromatin-associated RNA fractions isolated from control or U4 AMO treated cells. Values are normalised in each case to those obtained in the chromatin fraction of control AMO treated cells (given a value of 1). U4 AMO causes a substantial reduction in levels of chromatin-associated U4, U5 and particularly U6 snRNA while levels of U1 and U2 are less significantly altered. This is consistent with the U4 AMO impairing spliceosome assembly subsequent to recruitment of U2 snRNA.
* Denotes p<0.05

## Slide 6
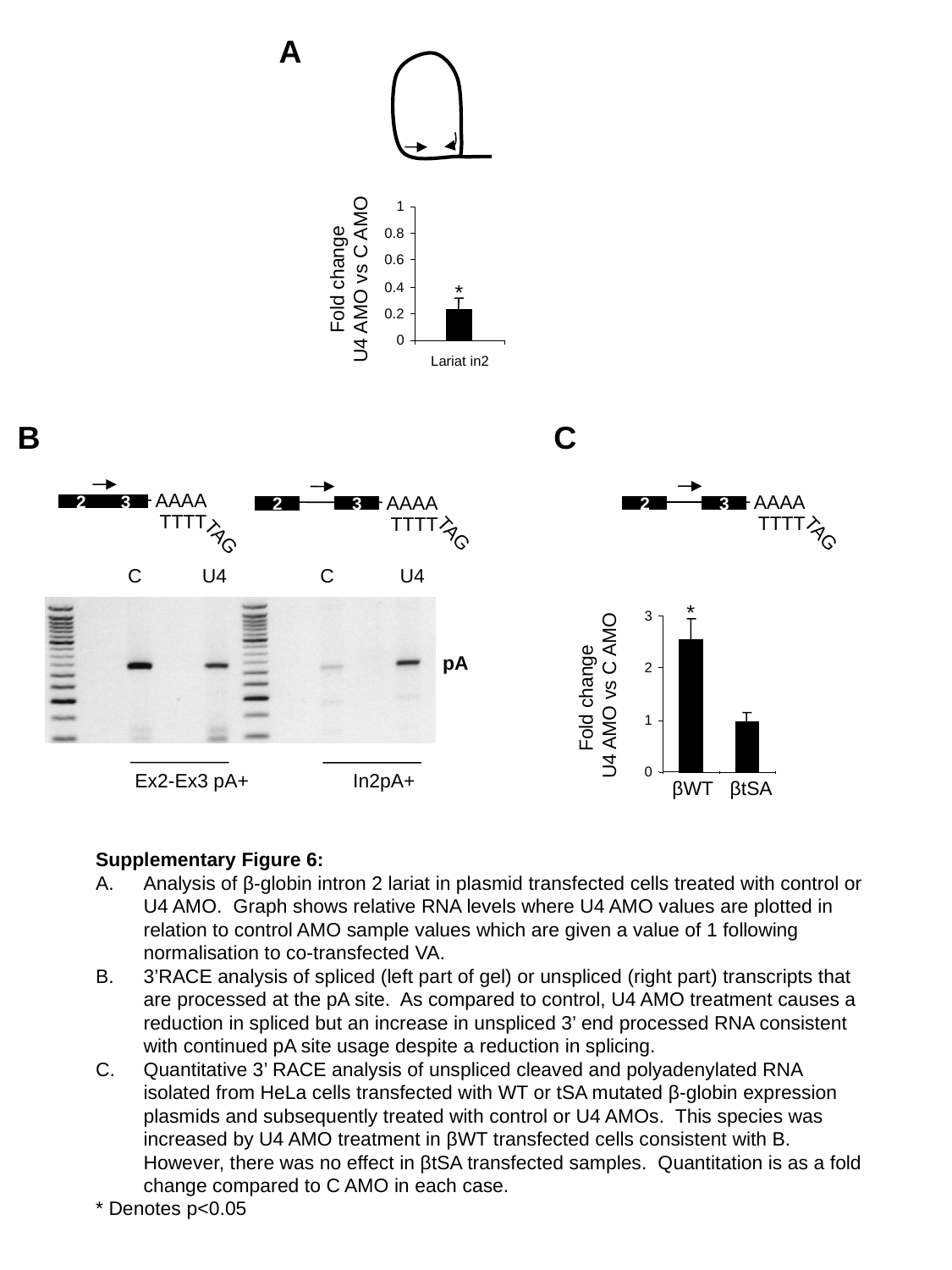

A
1
0.8
Fold change
U4 AMO vs C AMO
0.6
*
0.4
0.2
0
Lariat in2
B C
AAAA
AAAA
AAAA
3
2
3
3
2
2
TTTT
TTTT
TTTT
TAG
TAG
TAG
C U4 C U4
*
3
pA
2
Fold change
 U4 AMO vs C AMO
1
Ex2-Ex3 pA+ In2pA+
0
βWT βtSA
Supplementary Figure 6:
Analysis of β-globin intron 2 lariat in plasmid transfected cells treated with control or U4 AMO. Graph shows relative RNA levels where U4 AMO values are plotted in relation to control AMO sample values which are given a value of 1 following normalisation to co-transfected VA.
3’RACE analysis of spliced (left part of gel) or unspliced (right part) transcripts that are processed at the pA site. As compared to control, U4 AMO treatment causes a reduction in spliced but an increase in unspliced 3’ end processed RNA consistent with continued pA site usage despite a reduction in splicing.
Quantitative 3’ RACE analysis of unspliced cleaved and polyadenylated RNA isolated from HeLa cells transfected with WT or tSA mutated β-globin expression plasmids and subsequently treated with control or U4 AMOs. This species was increased by U4 AMO treatment in βWT transfected cells consistent with B. However, there was no effect in βtSA transfected samples. Quantitation is as a fold change compared to C AMO in each case.
* Denotes p<0.05

## Slide 7
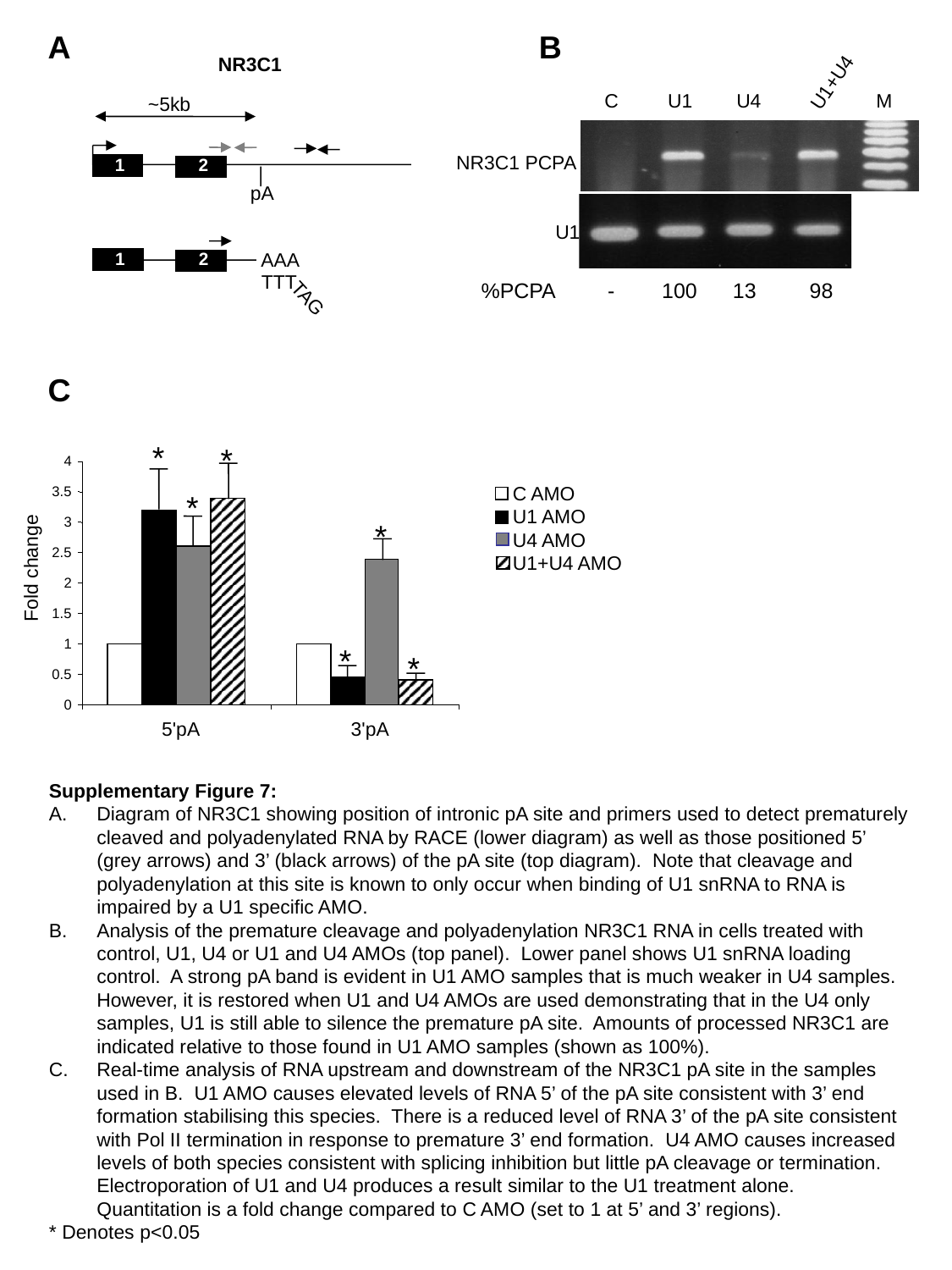

A B
C
NR3C1
U1+U4
C U1 U4 M
~5kb
NR3C1 PCPA
 U1
2
1
pA
AAA
2
1
TTT
TAG
%PCPA - 100 13 98
*
*
4
C AMO
U1 AMO
U4 AMO
U1+U4 AMO
*
3.5
*
3
2.5
Fold change
2
1.5
*
1
*
0.5
0
5'pA
3'pA
Supplementary Figure 7:
Diagram of NR3C1 showing position of intronic pA site and primers used to detect prematurely cleaved and polyadenylated RNA by RACE (lower diagram) as well as those positioned 5’ (grey arrows) and 3’ (black arrows) of the pA site (top diagram). Note that cleavage and polyadenylation at this site is known to only occur when binding of U1 snRNA to RNA is impaired by a U1 specific AMO.
Analysis of the premature cleavage and polyadenylation NR3C1 RNA in cells treated with control, U1, U4 or U1 and U4 AMOs (top panel). Lower panel shows U1 snRNA loading control. A strong pA band is evident in U1 AMO samples that is much weaker in U4 samples. However, it is restored when U1 and U4 AMOs are used demonstrating that in the U4 only samples, U1 is still able to silence the premature pA site. Amounts of processed NR3C1 are indicated relative to those found in U1 AMO samples (shown as 100%).
Real-time analysis of RNA upstream and downstream of the NR3C1 pA site in the samples used in B. U1 AMO causes elevated levels of RNA 5’ of the pA site consistent with 3’ end formation stabilising this species. There is a reduced level of RNA 3’ of the pA site consistent with Pol II termination in response to premature 3’ end formation. U4 AMO causes increased levels of both species consistent with splicing inhibition but little pA cleavage or termination. Electroporation of U1 and U4 produces a result similar to the U1 treatment alone. Quantitation is a fold change compared to C AMO (set to 1 at 5’ and 3’ regions).
* Denotes p<0.05

## Slide 8
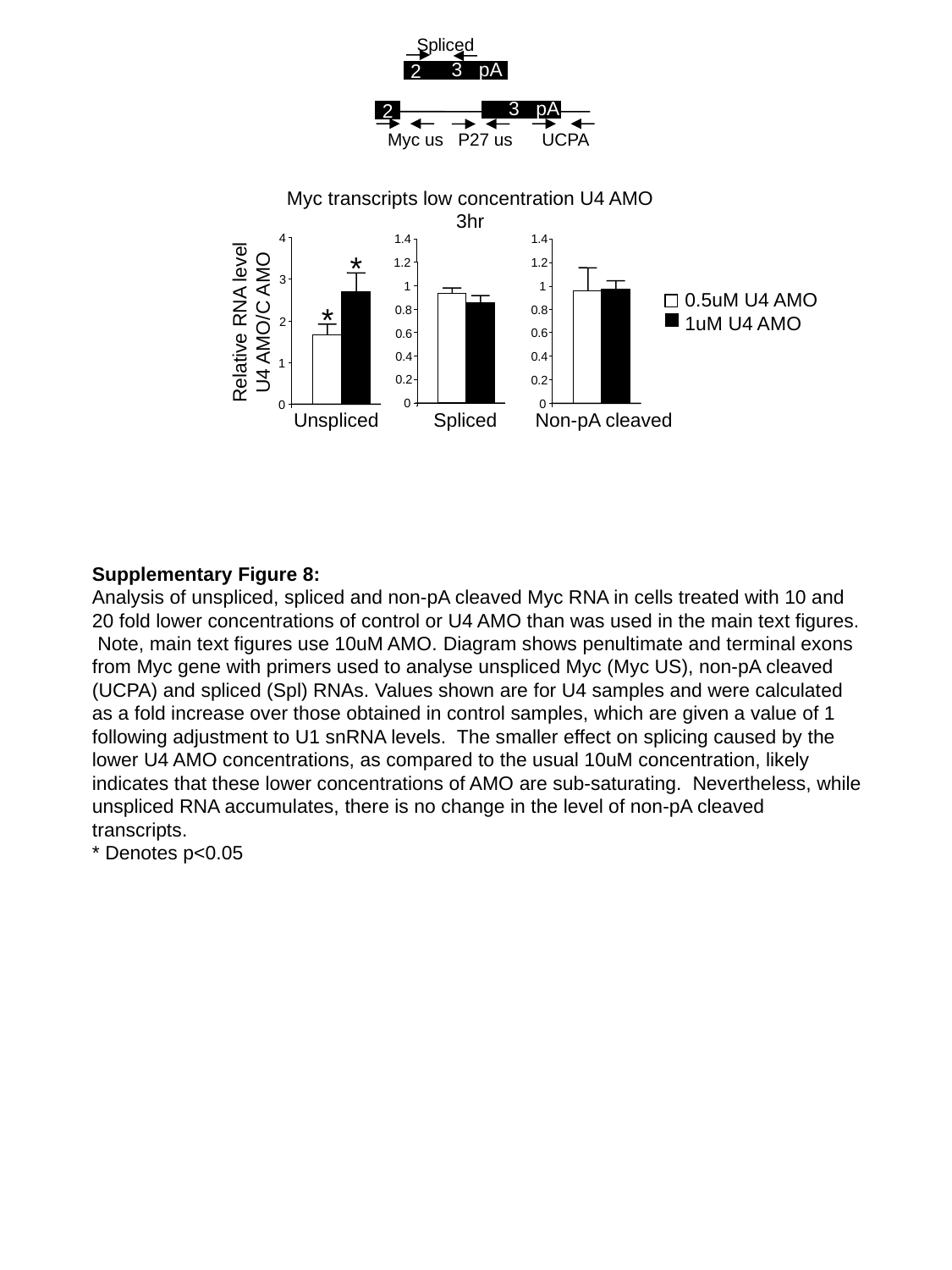

Spliced
3 pA
2
3 pA
2
Myc us P27 us UCPA
Myc transcripts low concentration U4 AMO
3hr
4
 1.4
1.4
*
 1.2
1.2
3
1
1
0.5uM U4 AMO
1uM U4 AMO
Relative RNA level
U4 AMO/C AMO
*
 0.8
0.8
2
0.6
0.6
0.4
0.4
1
0.2
0.2
0
0
0
Unspliced Spliced Non-pA cleaved
Supplementary Figure 8:
Analysis of unspliced, spliced and non-pA cleaved Myc RNA in cells treated with 10 and 20 fold lower concentrations of control or U4 AMO than was used in the main text figures. Note, main text figures use 10uM AMO. Diagram shows penultimate and terminal exons from Myc gene with primers used to analyse unspliced Myc (Myc US), non-pA cleaved (UCPA) and spliced (Spl) RNAs. Values shown are for U4 samples and were calculated as a fold increase over those obtained in control samples, which are given a value of 1 following adjustment to U1 snRNA levels. The smaller effect on splicing caused by the lower U4 AMO concentrations, as compared to the usual 10uM concentration, likely indicates that these lower concentrations of AMO are sub-saturating. Nevertheless, while unspliced RNA accumulates, there is no change in the level of non-pA cleaved transcripts.
* Denotes p<0.05

## Slide 9
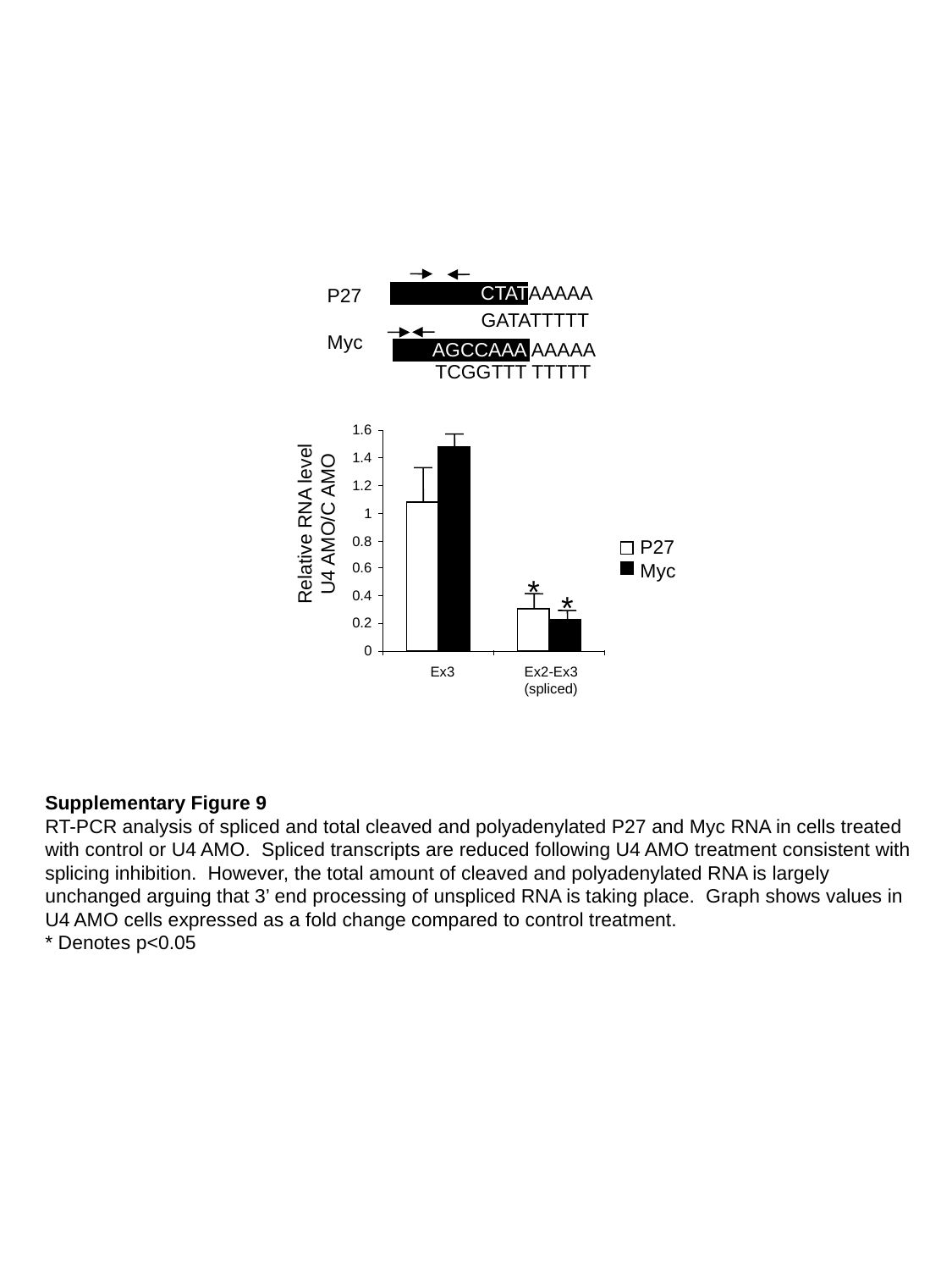

CTATAAAAA
P27
Myc
GATATTTTT
AGCCAAA AAAAA
TCGGTTT TTTTT
1.6
1.4
1.2
Relative RNA level
U4 AMO/C AMO
1
P27
Myc
0.8
0.6
*
*
0.4
0.2
0
Ex3
Ex2-Ex3
(spliced)
Supplementary Figure 9
RT-PCR analysis of spliced and total cleaved and polyadenylated P27 and Myc RNA in cells treated with control or U4 AMO. Spliced transcripts are reduced following U4 AMO treatment consistent with splicing inhibition. However, the total amount of cleaved and polyadenylated RNA is largely unchanged arguing that 3’ end processing of unspliced RNA is taking place. Graph shows values in U4 AMO cells expressed as a fold change compared to control treatment.
* Denotes p<0.05

## Slide 10
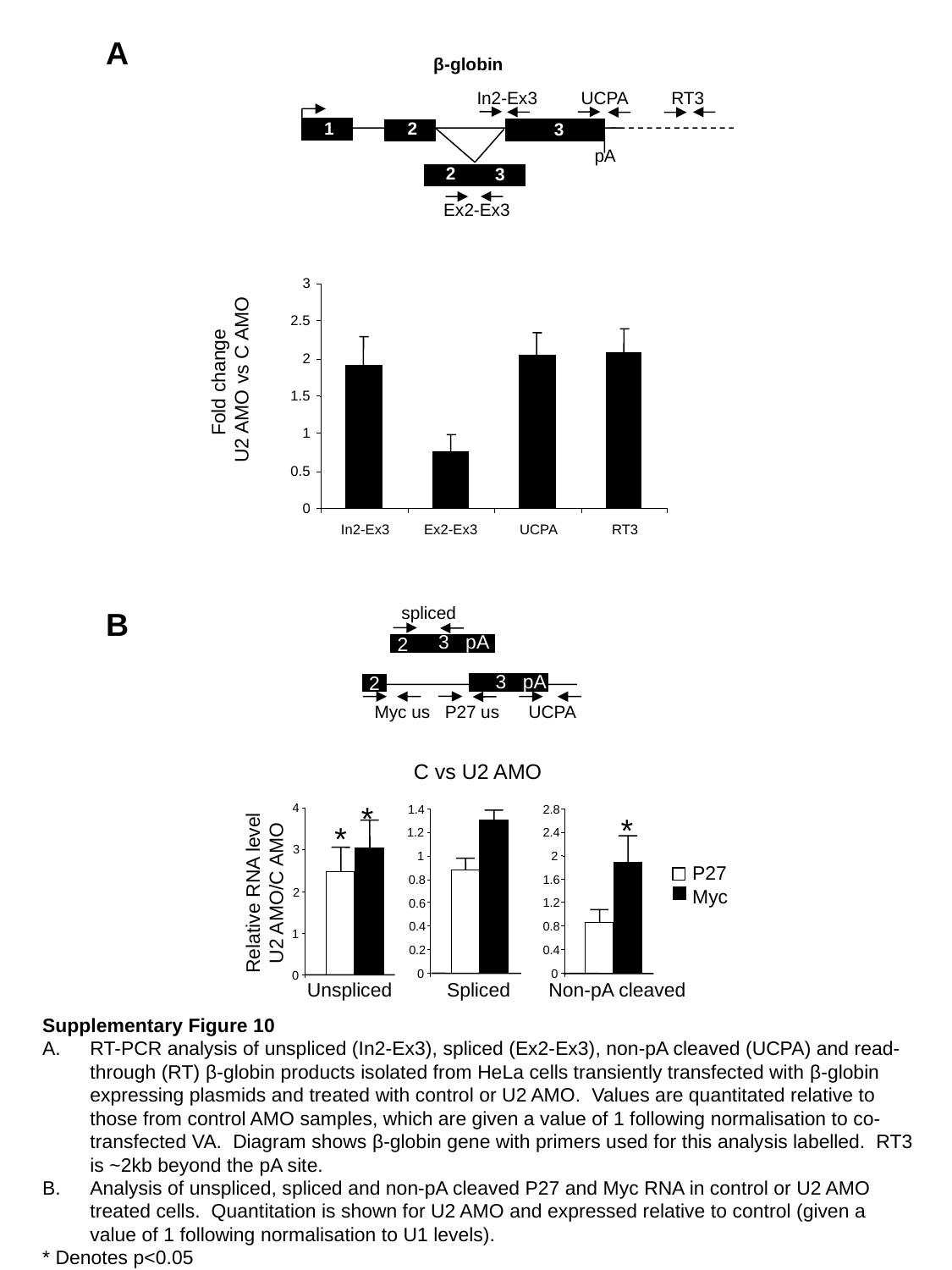

A
B
β-globin
In2-Ex3 UCPA RT3
3
2
1
pA
3
2
Ex2-Ex3
3
2.5
2
Fold change
 U2 AMO vs C AMO
1.5
1
0.5
0
In2-Ex3
Ex2-Ex3
UCPA
RT3
spliced
3 pA
2
3 pA
2
Myc us P27 us UCPA
C vs U2 AMO
*
4
2.8
 1.4
*
*
2.4
 1.2
3
2
1
P27
Myc
Relative RNA level
U2 AMO/C AMO
1.6
 0.8
2
1.2
0.6
0.8
0.4
1
0.4
0.2
0
0
0
Unspliced Spliced Non-pA cleaved
Supplementary Figure 10
RT-PCR analysis of unspliced (In2-Ex3), spliced (Ex2-Ex3), non-pA cleaved (UCPA) and read-through (RT) β-globin products isolated from HeLa cells transiently transfected with β-globin expressing plasmids and treated with control or U2 AMO. Values are quantitated relative to those from control AMO samples, which are given a value of 1 following normalisation to co-transfected VA. Diagram shows β-globin gene with primers used for this analysis labelled. RT3 is ~2kb beyond the pA site.
Analysis of unspliced, spliced and non-pA cleaved P27 and Myc RNA in control or U2 AMO treated cells. Quantitation is shown for U2 AMO and expressed relative to control (given a value of 1 following normalisation to U1 levels).
* Denotes p<0.05

## Slide 11
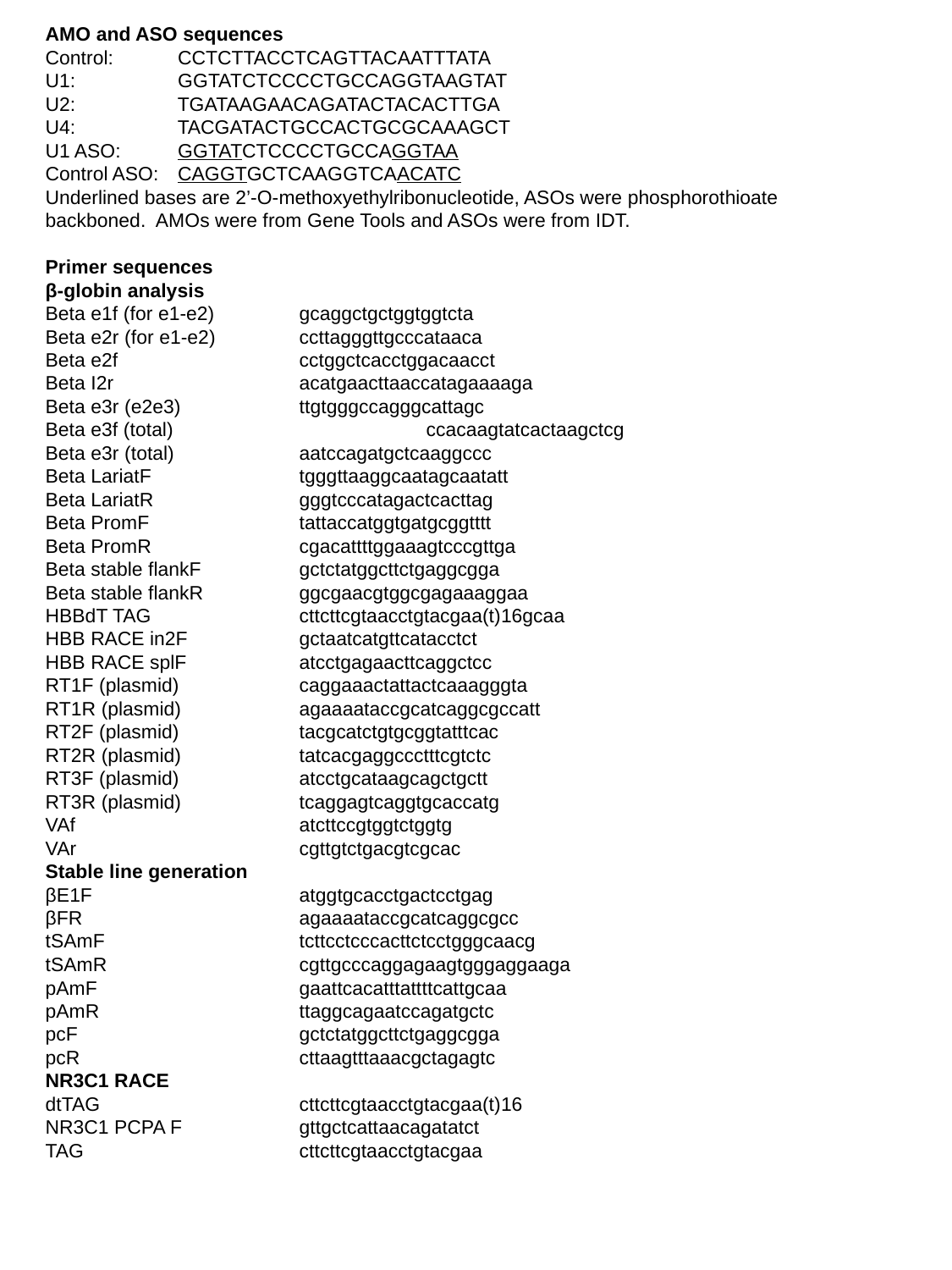

AMO and ASO sequences
Control:	 CCTCTTACCTCAGTTACAATTTATA
U1:	 GGTATCTCCCCTGCCAGGTAAGTAT
U2:	 TGATAAGAACAGATACTACACTTGA
U4:	 TACGATACTGCCACTGCGCAAAGCT
U1 ASO:	 GGTATCTCCCCTGCCAGGTAA
Control ASO:	 CAGGTGCTCAAGGTCAACATC
Underlined bases are 2’-O-methoxyethylribonucleotide, ASOs were phosphorothioate backboned. AMOs were from Gene Tools and ASOs were from IDT.
Primer sequences
β-globin analysis
Beta e1f (for e1-e2)	gcaggctgctggtggtcta
Beta e2r (for e1-e2)	ccttagggttgcccataaca
Beta e2f		cctggctcacctggacaacct
Beta I2r		acatgaacttaaccatagaaaaga
Beta e3r (e2e3)	ttgtgggccagggcattagc
Beta e3f (total)		ccacaagtatcactaagctcg
Beta e3r (total)	aatccagatgctcaaggccc
Beta LariatF		tgggttaaggcaatagcaatatt
Beta LariatR		gggtcccatagactcacttag
Beta PromF		tattaccatggtgatgcggtttt
Beta PromR		cgacattttggaaagtcccgttga
Beta stable flankF	gctctatggcttctgaggcgga
Beta stable flankR	ggcgaacgtggcgagaaaggaa
HBBdT TAG		cttcttcgtaacctgtacgaa(t)16gcaa
HBB RACE in2F	gctaatcatgttcatacctct
HBB RACE splF	atcctgagaacttcaggctcc
RT1F (plasmid)	caggaaactattactcaaagggta
RT1R (plasmid)	agaaaataccgcatcaggcgccatt
RT2F (plasmid)	tacgcatctgtgcggtatttcac
RT2R (plasmid)	tatcacgaggccctttcgtctc
RT3F (plasmid)	atcctgcataagcagctgctt
RT3R (plasmid)	tcaggagtcaggtgcaccatg
VAf		atcttccgtggtctggtg
VAr		cgttgtctgacgtcgcac
Stable line generation
βE1F		atggtgcacctgactcctgag
βFR		agaaaataccgcatcaggcgcc
tSAmF		tcttcctcccacttctcctgggcaacg
tSAmR		cgttgcccaggagaagtgggaggaaga
pAmF		gaattcacatttattttcattgcaa
pAmR		ttaggcagaatccagatgctc
pcF		gctctatggcttctgaggcgga
pcR		cttaagtttaaacgctagagtc
NR3C1 RACE
dtTAG		cttcttcgtaacctgtacgaa(t)16
NR3C1 PCPA F	gttgctcattaacagatatct
TAG		cttcttcgtaacctgtacgaa

## Slide 12
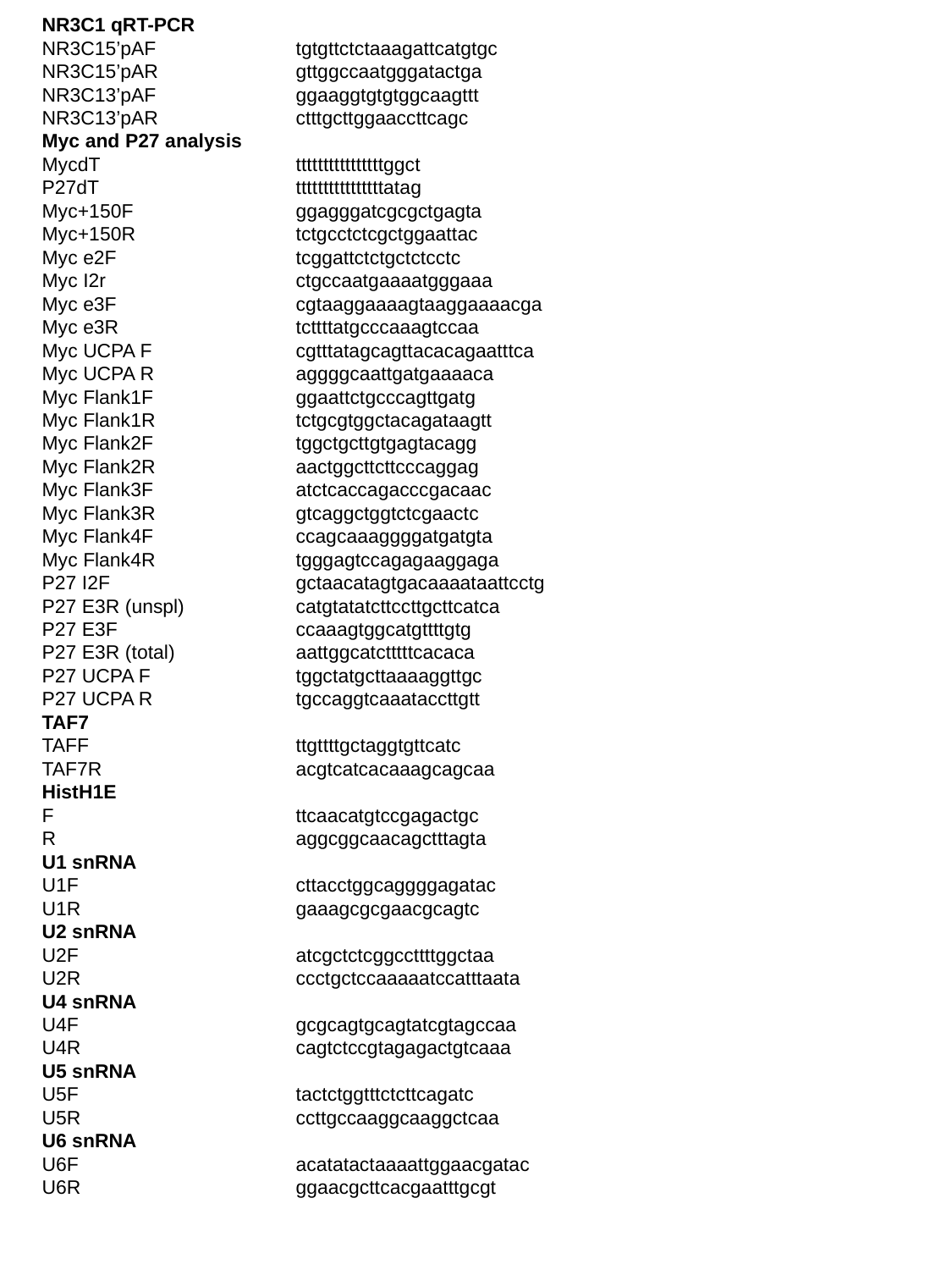

NR3C1 qRT-PCR
NR3C15’pAF		tgtgttctctaaagattcatgtgc
NR3C15’pAR		gttggccaatgggatactga
NR3C13’pAF		ggaaggtgtgtggcaagttt
NR3C13’pAR		ctttgcttggaaccttcagc
Myc and P27 analysis
MycdT		ttttttttttttttttggct
P27dT		ttttttttttttttttatag
Myc+150F		ggagggatcgcgctgagta
Myc+150R		tctgcctctcgctggaattac
Myc e2F		tcggattctctgctctcctc
Myc I2r		ctgccaatgaaaatgggaaa
Myc e3F		cgtaaggaaaagtaaggaaaacga
Myc e3R		tcttttatgcccaaagtccaa
Myc UCPA F		cgtttatagcagttacacagaatttca
Myc UCPA R		aggggcaattgatgaaaaca
Myc Flank1F		ggaattctgcccagttgatg
Myc Flank1R		tctgcgtggctacagataagtt
Myc Flank2F		tggctgcttgtgagtacagg
Myc Flank2R		aactggcttcttcccaggag
Myc Flank3F		atctcaccagacccgacaac
Myc Flank3R		gtcaggctggtctcgaactc
Myc Flank4F		ccagcaaaggggatgatgta
Myc Flank4R		tgggagtccagagaaggaga
P27 I2F		gctaacatagtgacaaaataattcctg
P27 E3R (unspl)	catgtatatcttccttgcttcatca
P27 E3F		ccaaagtggcatgttttgtg
P27 E3R (total)	aattggcatctttttcacaca
P27 UCPA F		tggctatgcttaaaaggttgc
P27 UCPA R		tgccaggtcaaataccttgtt
TAF7
TAFF		ttgttttgctaggtgttcatc
TAF7R		acgtcatcacaaagcagcaa
HistH1E
F		ttcaacatgtccgagactgc
R		aggcggcaacagctttagta
U1 snRNA
U1F		cttacctggcaggggagatac
U1R		gaaagcgcgaacgcagtc
U2 snRNA
U2F		atcgctctcggccttttggctaa
U2R		ccctgctccaaaaatccatttaata
U4 snRNA
U4F		gcgcagtgcagtatcgtagccaa
U4R		cagtctccgtagagactgtcaaa
U5 snRNA
U5F		tactctggtttctcttcagatc
U5R		ccttgccaaggcaaggctcaa
U6 snRNA
U6F		acatatactaaaattggaacgatac
U6R		ggaacgcttcacgaatttgcgt
