# Supplementary material for: Splicing-coupled 3′ end formation requires a terminal splice acceptor site, but not intron excision
Source: Nucleic Acids Res. 2013 May 28;41(14):7101–14. doi: 10.1093/nar/gkt446 (PMC3737548; doi:10.1093/nar/gkt446)
Supplement: Supplementary Data [file supp_gkt446_nar-00182-a-2013-File004.doc]

**Supplementary Materials and Methods**

**Chromatin and RNA Immunoprecipitation**

A confluent 10cm dish of HeLa or HEK cells were cross-linked in 1% formaldehyde for 10 minutes at room temperature. Cross-links were quenched in 125mM glycine, cells were rinsed in PBS and then collected by centrifugation (500xg for 5 mins). Cells were sonicated in 400μl RIPA buffer (150mM NaCl, 1% NP40, 0.5% DOC, 0.1% SDS, 50mM Tris.Cl pH8, 5mM EDTA pH8) (30s on, 30s off x 12mins on high in a bioruptor sonicator). Chromatin was clarified by centrifugation at 13000xg for 10mins and supernatants added to 20μl of protein A/G dynabeads (Invitrogen) pre- incubated in 500μl RIPA buffer for 2 hours with antibody (3μg N20, 3μg anti-CstF77). No antibody controls were performed in parallel. Following overnight rotation at 4^o^C, the beads were washed 2x in RIPA buffer, 4x in wash buffer (500mM NaCl, 1% NP40, 1% DOC, 100mM Tris.Cl pH8.5) and 2x in RIPA buffer. Immune complexes were eluted in 0.1M NaHCO_3_/1%SDS (15mins rotation at room temperature). Cross-links were reversed for 5 hours at 65^o^C (250mM NaCl and 1μg RNase A). DNA was phenol chloroform extracted and ethanol precipitated. Generally, 1/50^th^ was used for each real-time PCR reaction. For RNA immunoprecipitation, the same protocol was followed but immunoprecipitated material was treated for 1 hour with Turbo DNase prior to reverse transcription and real-time PCR analysis.

**S1 nuclease analysis**

End labelled probes for VA and β-globin were prepared by Klenow fill in reactions of the VA and β plasmids digested with Bam HI or Eco RI respectively. These were precipitated with 5-10μg of total RNA and resuspended in 30ul R-loop buffer (40mM PIPES pH7.4, 1mM EDTA, 400mM NaCl, 80% (v/v) formamide) before denaturation (85^o^C x 10mins) and overnight hybridisation (52^o^C x 16 hours). Samples were digested for 2 hours in 300μl S1 buffer (300mM NaCl, 50mM Na acetate, 4.5mM ZnSO_4_) at 30^o^C in the presence of 2μl S1 nuclease (Promega). Following ethanol precipitation, samples were resolved on 6% denaturing acrylamide gels.
